# Exosomes derived from palmitic acid-treated hepatocytes induce fibrotic activation of hepatic stellate cells

**DOI:** 10.1038/s41598-017-03389-2

**Published:** 2017-06-16

**Authors:** Young-Sun Lee, So Yeon Kim, Eunjung Ko, Jun-Hee Lee, Hyon-Seung Yi, Yang Jae Yoo, Jihye Je, Sang Jun Suh, Young Kul Jung, Ji Hoon Kim, Yeon Seok Seo, Hyung Joon Yim, Won-Il Jeong, Jong Eun Yeon, Soon Ho Um, Kwan Soo Byun

**Affiliations:** 1 Department of Internal Medicine, Korea University College of Medicine, Seoul, Korea; 2 Lab of Liver Research, Biomedical Science and Engineering Interdisciplinary Program, Korea Advanced Institute of Science and Technology (KAIST), Daejeon, 34141 Republic of Korea; 3 Graduate School of Medical Science and Engineering, Korea Advanced Institute of Science and Technology (KAIST), Daejeon, 34141 Republic of Korea; 4 Department of Internal Medicine, Chungnam National University School of Medicine, Daejeon, 305-764 Republic of Korea

## Abstract

Non-alcoholic fatty liver disease (NAFLD) is a dominant cause of chronic liver disease, but the exact mechanism of progression from simple steatosis to nonalcoholic steatohepatitis (NASH) remains unknown. Here, we investigated the role of exosomes in NAFLD progression. Exosomes were isolated from a human hepatoma cell line treated with palmitic acid (PA) and their miRNA profiles examined by microarray. The human hepatic stellate cell (HSC) line (LX-2) was then treated with exosome isolated from hepatocytes. Compared with controls, PA-treated hepatocytes displayed significantly increased CD36 and exosome production. The microarray analysis showed there to be distinctive miRNA expression patterns between exosomes from vehicle- and PA-treated hepatocytes. When LX-2 cells were cultured with exosomes from PA-treated hepatocytes, the expression of genes related to the development of fibrosis were significantly amplified compared to those treated with exosomes from vehicle-treated hepatocytes. In conclusion, PA treatment enhanced the production of exosomes in these hepatocytes and changed their exosomal miRNA profile. Moreover, exosomes derived from PA-treated hepatocytes caused an increase in the expression levels of fibrotic genes in HSCs. Therefore, exosomes may have important roles in the crosstalk between hepatocytes and HSCs in the progression from simple steatosis to NASH.

## Introduction

Nonalcoholic fatty liver disease (NAFLD) is becoming a leading cause of chronic liver disease, and induces liver cirrhosis and hepatocellular carcinoma (HCC)^1, 2^. Moreover, the incidence of NAFLD has been growing rapidly worldwide, becoming an increasingly important issue for personal and public health^3^. As a phased disease, NAFLD encompasses a broad spectrum of pathologies, from simple steatosis to steatohepatitis and cirrhosis^4^. Understanding the mechanisms that lead to NAFLD developing into the more serious nonalcoholic steatohepatitis (NASH) is an increasingly important issue. Both double and multi-hit models have been suggested to explain the pathogenesis of NAFLD^5, 6^. Insulin resistance and dysregulated lipid metabolism induce excessive fat accumulation in the liver, resulting in hepatic steatosis. In previous studies, several factors were introduced to explain NASH aggravation, such as gut-derived bacterial toxins, adiponectin imbalance, oxidative stress, activation of hepatic stellate cells (HSCs), and activation of pro-fibrotic and pro-inflammatory factors^7^. However, it is still unclear which factors are the most important in the development of the more aggressive diseases, such as NASH, cirrhosis, and HCC.

Exosomes are a type of small, extracellular vesicle that range in size from 30 nm to 100 nm. They contain various cellular molecules, such as proteins, mRNAs, and miRNAs^8^. Exosomes have critical roles in pathogenesis and can serve as biomarkers or therapeutic targets for various diseases, including liver disease^9^. In NAFLD, circulating extracellular vesicles can affect hepatic cells and are involved in intercellular signalling, tissue injury and repair, and matrix remodeling^10, 11^. Specific cargo molecules carried by exosomes are utilized for intercellular signal transduction. Among these molecules, small non-coding microRNAs (miRNA) have particularly important epigenetic functions, post-transcriptionally regulating gene expression. More than 21,000 miRNAs had been identified^12^ and several have been examined in NAFLD pathology^13^. However, it is still unclear whether miRNAs are involved in the progression of simple steatosis to NASH or NASH-related cirrhosis.

In addition to the various liver resident cells including hepatocytes, Kupffer cells (KC), HSCs, and liver sinusoidal endothelial cells, many immune cells regularly enter the liver through the hepatic artery, hepatic vein, and portal vein^14^. Cellular interactions between all of these cell populations are important in the pathogenesis and progression of liver diseases, and exosomes are crucial in inter-cellular transduction of these signals^15^. In alcoholic liver disease, the number of circulating exosomes is increased and exosomes released from hepatocytes also transduce miRNA signal to monocytes, resulting in their activation^16, 17^. In NAFLD, however, any inter-cellular signal transduction through exosomes and miRNA has not been examined.

In this study, we found that treatment with palmitic acid (PA) increased the production of exosomes and changed their miRNA profile. Furthermore, exosomes from PA-treated hepatocytes transduced a fibrosis-inducing signal to HSCs.

## Results

### Palmitic acid-treated cells produce more exosomes than vehicle-treated cells

**Figure 1:**
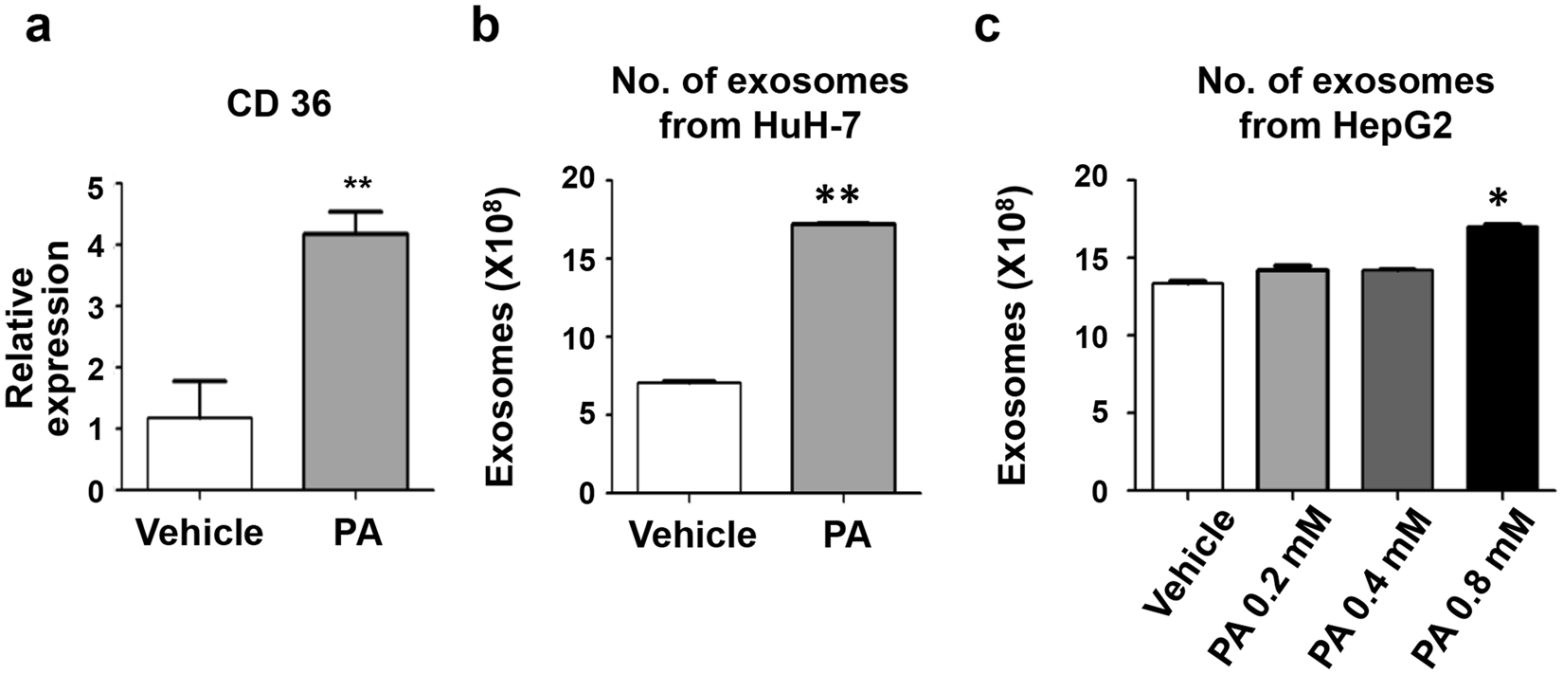
Palmitic acid treatment increases the production of exosomes in Huh7 and HepG2 cells. (**a**) CD36 expression levels were measured in Huh7 cells treated with either vehicle or palmitic acid (0.4 mM). (**b,c**) The total number of exosomes present in the supernatant from vehicle-treated or palmitic-acid treated Huh7 cells (**b**) and HepG2 cells (**c**). Data are expressed as the mean ± SEM of three independent experiments. **P < 0.01 compared with the corresponding control.

To induce lipid accumulation in hepatocytes, we cultured Huh7 cells with PA *in vitro*. The expression of CD36, a fatty acid translocase, was analysed in vehicle-treated Huh7 and PA-treated Huh7 cells. PA-treated Huh7 cells exhibited increased expression of CD36 compared to vehicle-treated Huh7 cells, demonstrating that PA treatment effectively impacted hepatocytes (Fig. 1a). Next, we analysed the number of isolated exosomes from vehicle- and PA-treated Huh7 cells to examine whether PA treatment changes hepatocyte exosome production. Interestingly, PA-treated Huh7 cells produced a greater number of exosomes than vehicle-treated Huh7 cells (17.2 × 10^8^ *vs*. 7.1 × 10^8^, P < 0.001) (Fig. 1b). HepG2 cells were also treated with a range of PA concentrations to assess if exosome production was dose-dependent. Exosome production was increased at all levels of PA tested, although only a PA concentration of 0.8 mM was found to be significant (Fig. 1c). These results indicate that PA treatment induces lipid accumulation in hepatocytes and increases production of exosomes.

### Characterization of exosome

**Figure 2:**
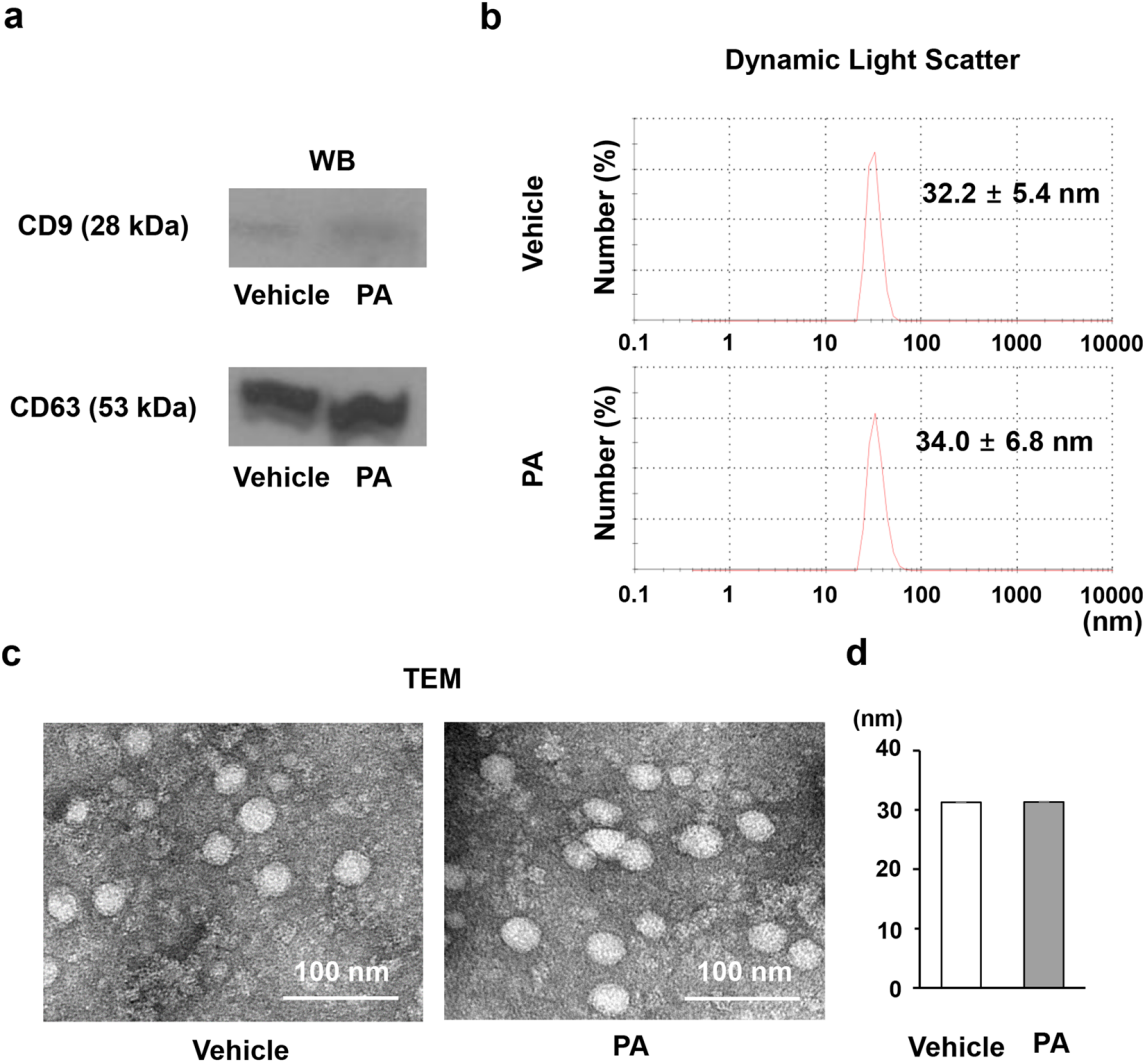
Characterization of exosomes. (**a**) Expression levels of CD9 (28 kDa) and CD63 (53 kDa) were determined by Western blotting. Protein was extracted from exosomes isolated from Huh7 cultured media. (**b**) The sizes of isolated exosomes were analyzed using dynamic light scattering. Each analysis was performed 30 times and data are expressed as the mean ± SD. (**c**) Exosomes visualized by TEM. The scale bar represents 100 nm. (**d**) The average size of exosomes was measured and data are expressed as the mean ± SD.

Several tools were used to characterize the exosomes from vehicle-treated cells and PA-treated cells, including Western blotting, dynamic light scatter size determination, and TEM (Fig. 2a–c). Both CD9 and CD63 are well known as exosomal markers and their expression was analysed by Western blotting; CD9 and CD63 were expressed at similar levels in both exosomes from vehicle-treated cells and PA-treated cells (Fig. 2a). Since exosomes range in size from 30 nm to 100 nm^8^, it is of interest to examine their average size. Using dynamic light scatter system we assessed the average size of exosomes isolated from treated hepatocytes. The mean size of the exosomes isolated from vehicle-treated cells was 32.2 ± 5.4 nm and was 34.0 ± 6.8 nm from PA-treated cells (Fig. 2b), with no significant difference being noted. Finally, the exosomes were characterized using TEM, which demonstrated that the exosomes size were similar in both groups (31.27 ± 2.96 nm in the vehicle-treated group vs. 31.34 ± 3.34 mm in the PA-treated group, P = 0.466) (Fig. 2c,d) again with no significant difference being noted. These findings show that exosomes from PA treated hepatocytes can be efficiently isolated and they are similar in size to those from vehicle-treated cells.

### PA treatment changes the pattern of miRNA expression in exosomes from hepatocytes

**Figure 3:**
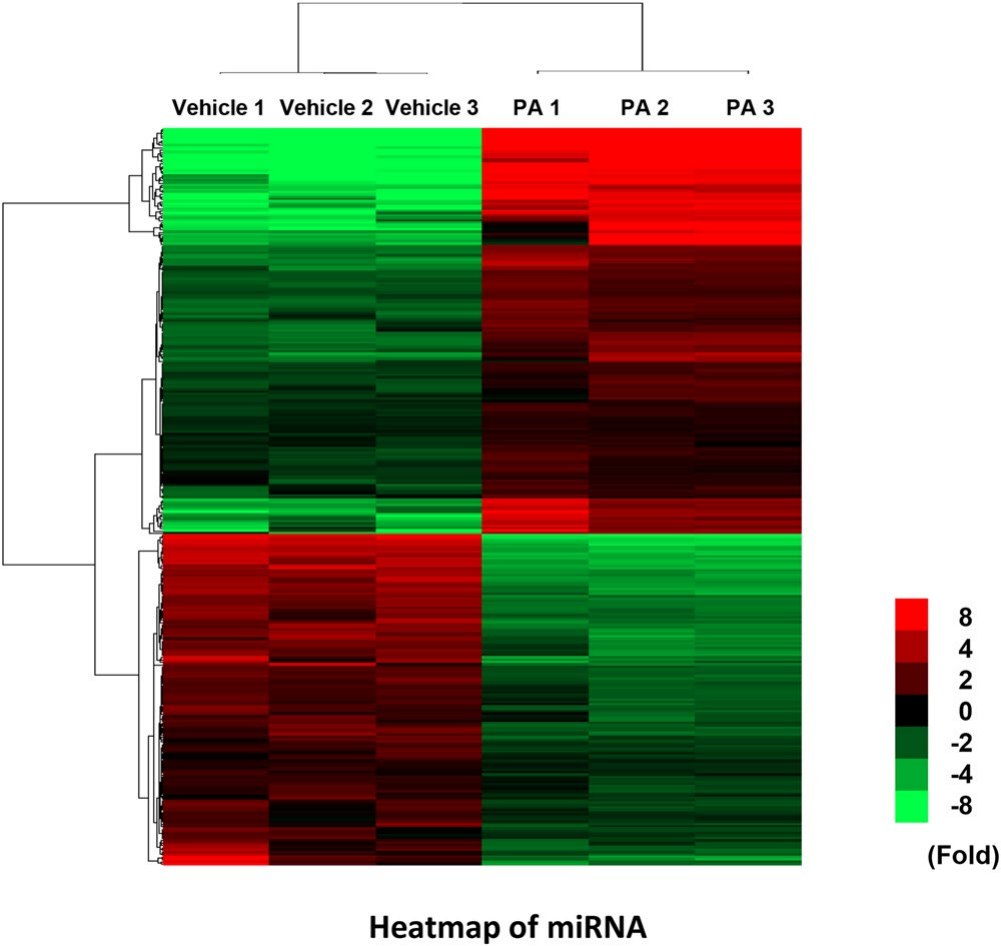
Expression profile of miRNA in exosomes. The heat map shows miRNAs that were significantly changed more than two-fold (P < 0.05) in exosomes from palmitic acid (PA)-treated Huh7 cells compared to exosomes from vehicle-treated Huh7 cells. The left-hand three columns represent the individual miRNA profiles of exosomes from three replicates of vehicle-treated Huh7 cells, whereas the right-hand three columns represent the miRNA profiles of exosomes from three replicates of PA-treated Huh7 cells. The right color bars represent the miRNA expression fold changes; The red color indicates that expression of the miRNA increased in exosomes from the PA-treated group compared to the vehicle-treated group, whereas green indicates that expression of the miRNA decreased in exosomes from the PA-treated group compared to those from the vehicle-treated group.

**Figure 4:**
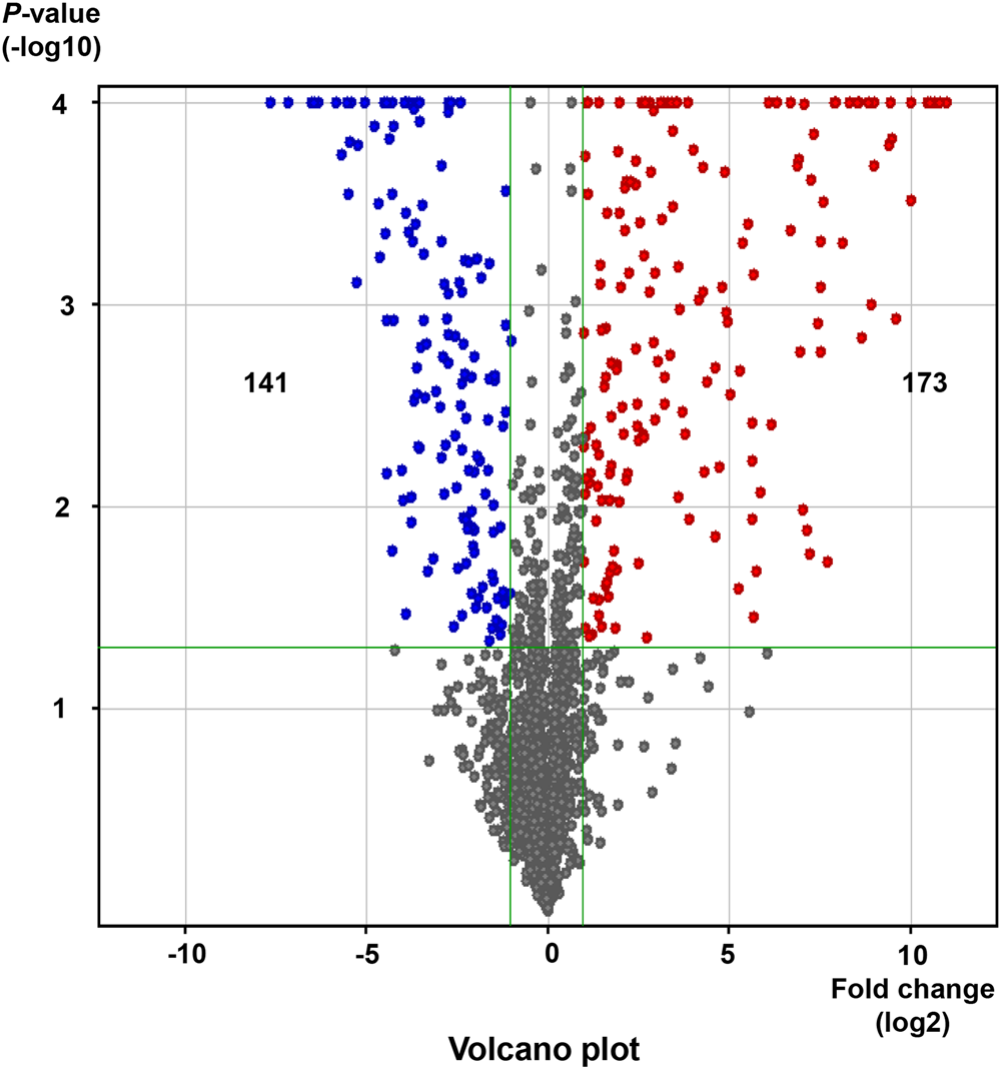
Volcano plot of miRNA in exosomes. The volcano plot shows the intensity of miRNA expression between exosomes from PA-treated Huh7 cells and exosomes from vehicle-treated Huh7 cells. The horizontal axis represents the log2 ratio and the vertical axis represents −log10 (t-test, P value). The red color indicates that the expression of the miRNA significantly increased more than two-fold in exosomes from the PA-treated group compared to those from the vehicle-treated group. The blue color indicates that the expression of the miRNA significantly decreased more than two fold in exosomes from the PA-treated group compared to those from the vehicle-treated group.

We used a microarray to compare the miRNA profiles of exosomes isolated from vehicle-treated Huh7 cells and PA-treated Huh7 cells. A total 2578 miRNAs were detected in isolated exosomes. As shown in Fig. 3, the miRNA heat map reveals that the 314 miRNA expression pattern in exosomes was significantly different between the vehicle- and PA-treated groups. A change in the expression of a miRNA with more than a two-fold increase or decrease with a P-value < 0.05, miRNA was considered to be a significant change. The expression of 173 miRNAs was significantly increased in the exosomes from the PA-treated group, whereas the expression of 141 miRNAs was significantly increased in the exosomes from the vehicle-treated group (Fig. 4).

**Figure 5:**
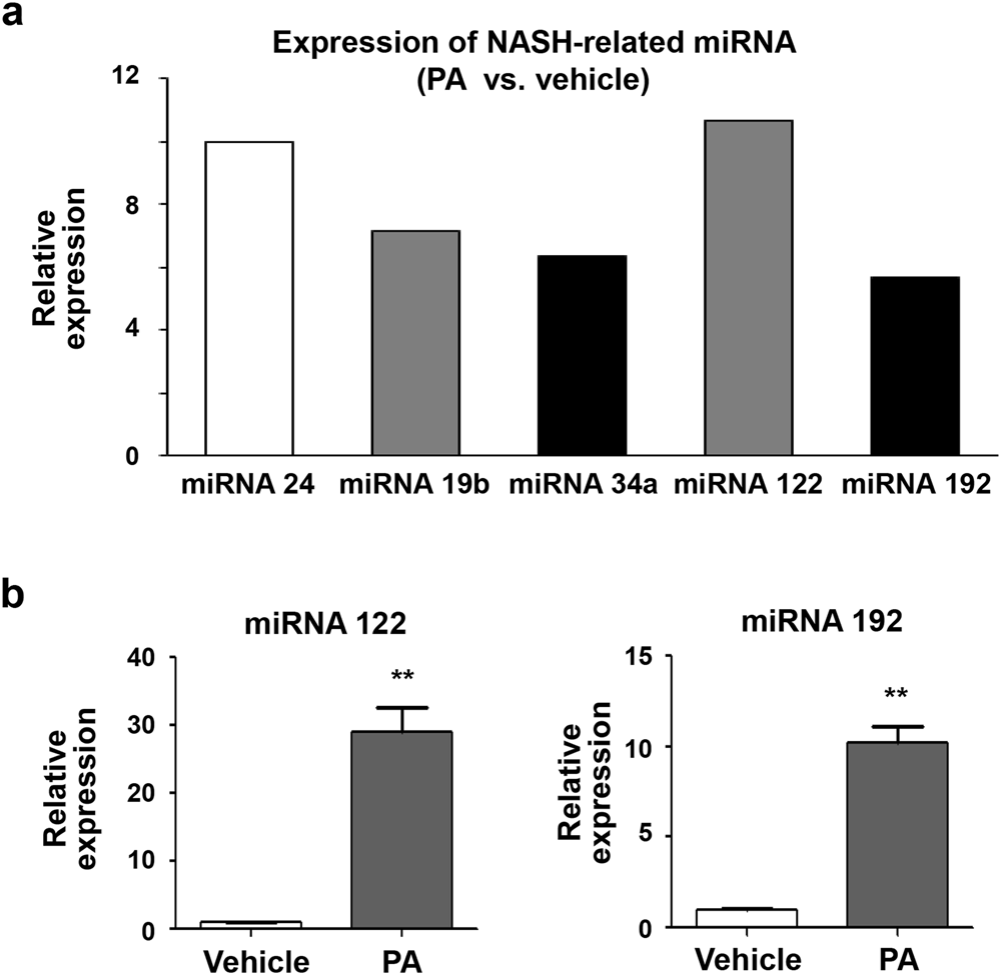
PA treatment changes expression of miRNA in hepatocytes. (**a**) The expression profiles of selected miRNAs associated with NASH. (**b**) The expression levels of miRNA 122 and miRNA 192 were validated using real-time PCR. **P < 0.01 compared with the corresponding control.

We specifically examined several miRNAs already known to be involved in NAFLD and NASH (Fig. 5a). The expression of the miRNAs 24^18^, 19b^19^, 34a^20^, 122^21^, and 192^22^ increased more than five-fold in exosomes from the PA-treated Huh7 cells, compared to those from vehicle-treated Huh7 cells. We next examined the expression levels of miRNA 122 and miRNA 192 using real-time PCR. As expected, exosomes from PA-treated Huh7 cells exhibited a 10-fold increase in the expression of miRNA 122 and miRNA 192 compared to vehicle-treated Huh7 cells (Fig. 5b). Taken together, PA treatment significantly alters the pattern of exosome production in hepatocytes and also increases the abundance of NASH-related miRNAs in exosomes released from PA-treated hepatoma cells.

### Exosomes derived from PA-treated hepatocytes and miRNA 192 enhance the expression of fibrosis markers in hepatic stellate cells

**Figure 6:**
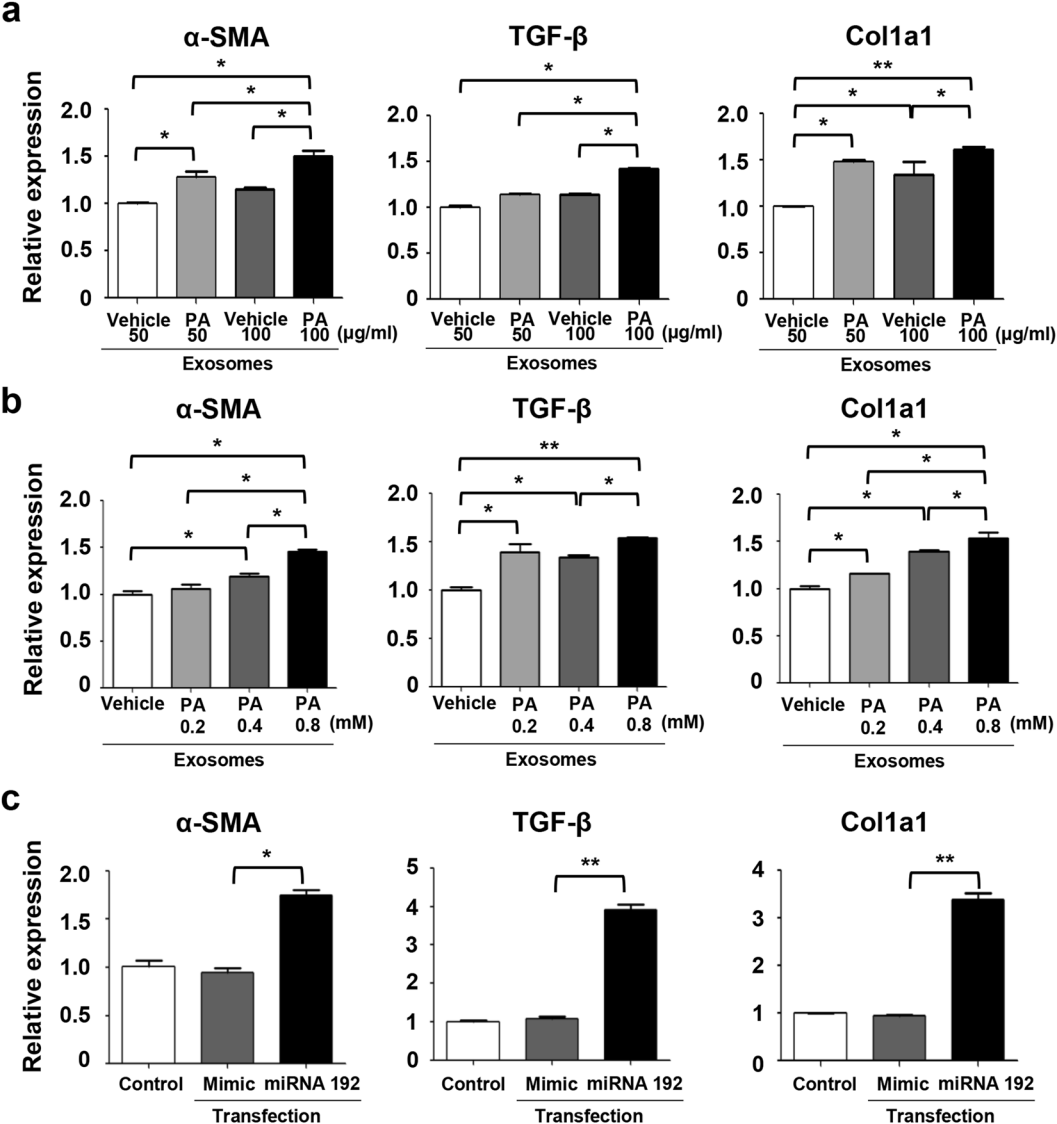
Exosomes derived from PA-treated Huh7 cells enhance expression of fibrosis markers in LX2 cells. (**a**) LX-2 cells were exposed to exosomes isolated from vehicle-treated Huh7 cells or PA-treated (0.4 mM) Huh7 cells. Each isolated exosome was added to the LX-2 cells at a concentration of 50 or 100 μg/mL. The expression levels of α-SMA, TGF-β, and Col1a1 were measured in LX-2 cells using real-time PCR. (**b**) LX-2 cells were exposed to isolated exosomes (50 μg/ml) from vehicle-treated Huh7 cells or PA-treated Huh7 cells at various concentrations (0.2 mM, 0.4 mM, and 0.8 mM). The expression of α-SMA, TGF-β, and Col1a1 were measured in LX-2 cells using real-time PCR. **(c)** LX-2 cells were transfected with miRNA 192 or a mimic control. The expression of α-SMA, TGF-β, and Col1a1 was measured in LX-2 cells using real-time PCR. *P < 0.05, **P < 0.01 compared with the corresponding control.

We next examined if exosomes from vehicle-treated hepatocytes and PA-treated hepatocytes can regulate the function of HSCs, specifically in relation to the development of fibrosis. After exposing HSCs to exosomes, several fibrosis-associated markers were analysed, including α-SMA, TGF-β, and Col1a1. Exosomes from PA-treated hepatocytes demonstrated an amplified expression of fibrosis markers in HSCs compared to those from vehicle-treated hepatocytes (Fig. 6a). Moreover, exosomes treatment with 100 μg/ml of concentration more triggered fibrosis marker than it with 50 μg/ml of concentration.

LX-2 cells were then exposed to exosomes from vehicle-treated hepatocytes or from hepatocytes treated with various concentration of PA (0.2 mM, 0.4 mM, and 0.8 mM). The expression of α-SMA, TGF-β, and Col1a1 increased in LX-2 cells exposed to exosomes from PA-treated hepatocytes, compared to exosomes from vehicle-treated hepatocytes. With the exception of TGF-β, the magnitude of the increase in expression for most transcripts correlated with PA treatment concentration (Fig. 6b). Although exosomes from the 0.2 mM PA-treated group showed an increased expression of TGF-β compared to the 0.4 mM PA-treated group, in general there was a similar dose dependent pattern. Repetitive treatment of LX-2 cells with exosomes showed similar results, which the expression of TGF-β and Col1a1 being increased significantly in LX-2 cells exposed to exosomes from PA-treated hepatocytes compared to exosomes from vehicle-treated hepatocytes; this was not seen for α-SMA (Supplementary Fig. 1). When HSCs from mice were treated with exosomes derived from treated mouse hepatocytes, the exosomes from PA-treated hepatocytes increased the HSC gene expression levels of α-SMA, TGF-β, and Col1a1 and the protein expression of TGF- β, compared to those exosomes from vehicle-treated hepatocytes (Supplementary Fig. 2a,b). It is worth nothing that the cell morphology of the HSCs was similar between two treatment groups (Supplementary Fig. 2c). Collectively, these data suggest that exosomes derived from PA-treated hepatocytes enhance expression of fibrosis markers in HSCs.

To explore whether this fibrotic behaviour of HSCs was due to the presence of the miRNAs in the exosomes, we directly transfected cells with miRNA 192. HSCs transfected with miRNA 192 showed enhanced expression of α-SMA, TGF-β, and Col1a1 compared to the negative control miRNA transfected cells (Fig. 6c).

Advanced stage NAFLD patients showed distinct number of exosomes and expression of miRNA in exosomes from sera compared to early stage NAFLD patients.

**Figure 7:**
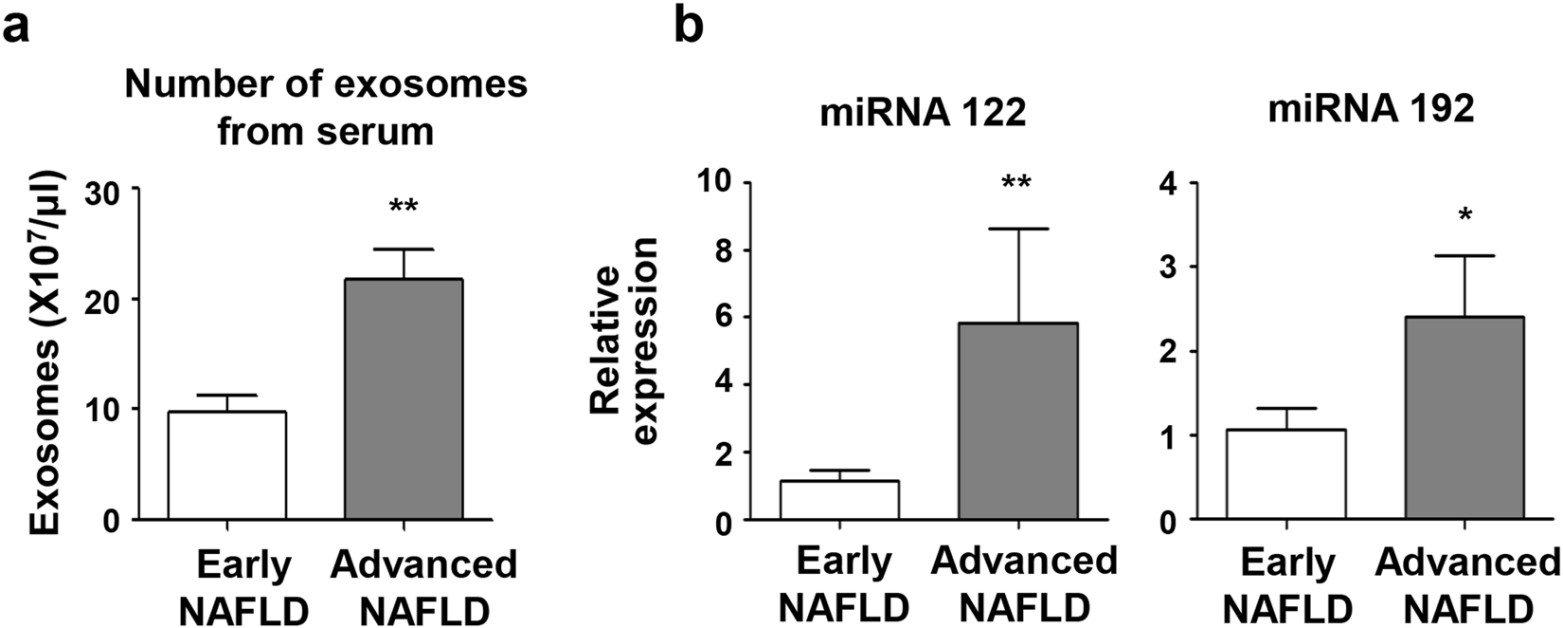
Number of exosomes in the sera in patients with NAFLD and the expression of miRNA 122 and miRNA 192 in exosomes. (**a**) The total number of exosomes was measured in sera from early stage (n = 3) or advanced stage (n = 3) NAFLD patients. (**b**) The expression of miRNA 122 and miRNA 192 were analysed using real-time PCR. Data are expressed as the mean ± SEM. *P < 0.05, **P < 0.01 compared with the corresponding control.

We next examined circulating exosome number in sera from six patients with biopsy-proven NAFLD; three of these patients had early stage NAFLD with S0 or S1 steatosis and F0 or F1 fibrosis (Supplementary Fig. 3a) and the remaining three patients had advanced stage NAFLD with S2–3 steatosis and F2–F4 fibrosis (Supplementary Fig. 3b). The number of exosomes significantly increased in the sera from the advanced stage NAFLD group compared to those from the early stage NAFLD group (21.73 ± 4.85 × 10^7^/μL *vs*. 9.75 ± 2.65 × 10^7^/μL, P < 0.001) (Fig. 7a). In addition, the expression of miRNA 122 and miRNA 192 were increased in circulating exosomes from the advanced stage NAFLD group compared to those from the early stage NAFLD group (Fig. 7b).

## Discussion

Determining the exact mechanisms that lead to NAFLD and NASH is important for disease identification and the development of therapeutics. Although insulin resistance and dysregulated lipid metabolism are known to be involved in NAFLD development and progression to NASH, factors that regulate fibrosis and its involvement in NASH are largely unknown^7^. In this study, we found that PA treatment of hepatocytes increases the production of exosomes and changes the miRNA profile of the exosomes. Furthermore, exosomes from PA-treated hepatocytes can increase the expression of fibrosis markers in HSCs.

Exosomes are small (30 to 100 nm) extracellular vesicles that are involved in various liver disease pathologies, including drug-induced liver injury, alcoholic liver disease, NAFLD, viral hepatitis, liver fibrosis and cirrhosis, and HCC^8, 9^. Exosomes can serve as useful biomarkers for diagnosis and prognosis, but may also serve as therapeutic target in various liver disease^15^. The role of exosomes in NAFLD is not known due to the limited number of studies that have been performed. However, it is known that production of exosomes and other extracellular vesicles are increased during NAFLD^23^, and intra-vesicular protein expression is altered^24^. Hirsova *et al*. demonstrated that extracellular vesicle formation can be induced by lipid treatment of macrophages to generate an inflammatory response^25^. Similarly, in our study, PA-treated hepatocytes exhibited increased exosome production and the contents of these exosomes were changed, likely due to lipid accumulation induced by the PA treatment. The increased production of exosomes may reflect disease severity and could potentially be used as a biomarker for NAFLD^23^. In our study, treatment with PA increased production of exosomes, so an estimation of exosome number may serve as an important measurement for NAFLD progression.

The most important function of exosomes is the conveyance of inter-cellular signalling molecules^26^. Exosomes are produced from intra-luminal vesicles of the multi-vesicular body, formed from the cell membrane and endosomes. During formation, exosomes incorporate cell- and disease-specific molecules, resulting in the transduction of pathologic signals^8^. They can be found in most body fluids, including serum, bile, saliva, urine, semen, and extracellular fluids^27^. Through this extracellular flow, exosomes migrate to adjacent or distant recipient cells and integrate into target cells either through receptors or by direct fusion with cellular membranes^15^. During liver disease, hepatocytes and non-parenchymal cells, including HSCs, cholangiocytes, liver sinusoidal endothelial cells, and other immune cells, may not only be the origin of exosomes but are also potentially targets themselves^28–30^. In this study, we found that exosomes mediate a fibrosis-inducing signal from lipid-accumulated hepatocytes to HSCs.

We used a variety of different methods to characterize the isolated exosomes. We performed Western blots to demonstrate the presence of CD9 and CD63, previously known markers of exosomes^8, 31, 32^. Using a dynamic light scattering system, the exosome size was determined to be approximately 30 nm. Finally, isolated exosomes were directly visualized using TEM. Through these technique, we fully identified and characterized the isolated exosomes and this information could be useful for further experiments. An accurate quantification of exosomes is a hurdle to that needs to be overcome for further investigation. Here, we calculated the number of isolated exosomes using an exosome quantitation kit that utilizes acetylcholinesterase activity^33^. After confirming and characterizing exosomes in our experimental system, we investigated the role these exosomes may play in conveying messages between hepatocytes and HSCs.

Exosomes contain a variety of cargo and cellular signals that can be transported between donor cells and recipient cells^9, 34^. Typical exosomal cargos consist of protein, lipid, and several nucleic acids, including mRNAs, miRNAs, and other non-coding RNAs^27^. Of particular importance here, miRNAs are RNA sequences of approximately 22 nucleotides that have a key role in regulating gene function binding target mRNAs^35, 36^. Although more than 21,000 miRNAs have been identified^12^, many of their functions remain unknown. Recently, miRNAs have been investigated as potential diagnostic and prognostic biomarker s for NAFLD, and as novel therapeutic targets. In both animal NAFLD models and human patients with NAFLD, miRNA expression profiles are significantly different between NASH and simple steatosis^21, 37^. Our *in vitro* model also demonstrated that PA treatment alters the global exosome miRNA expression patterns, including the expression of specific miRNAs known to contribute to the progression of simple steatosis to NASH, cirrhosis, and HCC^38^. In some cases, a 10-fold difference in miRNA expression was observed between exosomes from vehicle- and PA-treated hepatocytes.

Becker *et al*. have investigated the potential these miRNAs have as biomarkers during NASH^22^. They found that serum levels of miRNA −122, −192, and −21 were significantly different in patients with different NAFLD activity scores. In the present study, we also found that the expression of certain miRNAs increased in exosomes from PA-treated Huh7 cells compared to those from vehicle-treated Huh7 cells. Our real-time PCR validation analysis confirmed there was increased expression of miRNA 122, known to be one of the most abundant miRNAs in the liver. miRNA 192, which is associated with NASH progression and fibrosis, was also more abundant in exosomes from PA-treated cells^38, 39^. Moreover, we found that the expression of miRNA 122 and miRNA 192 were increased in circulating exosomes from advanced stage NAFLD patients compared to those from early stage NAFLD patients. Therefore, this miRNA expression in circulating exosomes might be useful as a biomarker for the diagnosis of advanced NAFLD or NASH.

HSCs have critical roles in the progression of fibrosis in various liver disease^40, 41^. When hepatic steatosis develops, HSCs are activated and express several fibrosis markers *in vitro*, such as TGF-β, TIMP-1, TIMP-2, and matrix-metallo-proteinase-2^42^. It has also been demonstrated that conditioned media from PA-treated hepatocytes can activate HSCs and induce fibrosis-associated signals, although the potential mechanisms have not been discovered. Our investigation of exosomes and their role in fibrotic activation of HSCs during hepatic steatosis suggests that exosomes are key mediators between HSCs and hepatocytes. PA-treated hepatocytes showed increased exosome production and an altered miRNA profiles that results in the regulation of fibrosis marker genes in HSCs. miRNAs are known to be associated with activation, apoptosis, collagen production, and proliferation of HSCs^43^. In this study, we found that direct transfection of miRNA 192 into HSCs increased the expression of fibrosis marker genes in HSCs. Therefore, miRNA profiling may be a useful representative marker to describe the activation status of HSCs at different stage of fibrosis in NASH.

In conclusion, our study found that PA treatment accelerated the production of exosomes in hepatocytes and altered their miRNA profile. In addition, exosomes originating from PA-treated hepatocytes induced the increased expression of fibrotic genes in HSCs. Therefore, exosomes may have important roles in the crosstalk between hepatocytes and HSCs during the progression from simple steatosis to NASH.

## Materials and Methods

### Hepatoma cell-lines and palmitic acid treatment

Huh7 and HepG2 cells were used as hepatoma cell lines. Huh7 and HepG2 cells were cultured in high glucose DMEM with 10% exosome-depleted foetal bovine serum (FBS) (Systemic Bioscience, Palo Alto, CA, USA) and 1% penicillin/streptomycin (Gibco, New York City, NY, USA). To induce lipid accumulation in hepatocytes, Huh7 and HepG2 cells were treated with palmitic acid (PA) (Sigma Aldrich, St. Louis, MO, USA) for eight hours at various concentrations (0.2, 0.4, and 0.8 mM). After collection of media for exosome analysis, cells were harvested with trypsin-EDTA (Gibco) and subjected to real-time PCR.

### Patients

Sera were collected from six patients with biopsy-proven NAFLD from November 2016 to January 2017. All patients had no other chronic liver disease including chronic viral hepatitis B/C, autoimmune hepatitis, primary biliary cholangitis, or alcoholic liver disease. Sera were stored at −20 °C and thawed for isolation of the exosome. All patients were agreed for experiment and publication using their tissues and sera with written informed consent. This study was approved by the institutional review board of Korea University Guro hospital (KUGH16089-001). All methods were performed in accordance with relevant guidelines and regulations.

### Exosome isolation and counting

Exosomes were isolated from cell culture supernatants using an exosome isolation kit (ExoQuick-TC^tm^, Systemic Biosciences), following the manufacturer’s protocol. After three rounds of serial filtration with 0.8, 0.45, and 0.2 μm filters (Corning, Coring, NY, USA), culture media samples were centrifuged at 3000 *g* for 15 minutes to remove cells and debris. The supernatant was transferred to sterile tubes and an exosome precipitation solution was added at a 5:1 ratio. Samples were mixed and left for 12 hours at 4 °C. Samples were then centrifuged at 1500 *g* for 30 minutes and supernatant carefully removed. The precipitated exosome pellets were re-suspended with PBS and either used immediately or stored at −80 °C until required. For quantification of isolated exosomes, acetylcholinesterase activity assay was performed using Exocet (Systemic Bioscience)^33^. Isolated exosomes were re-suspended with PBS and lysed with lysis buffer. Each sample was incubated at 37 °C for 5 minutes and mixed with reaction buffer in 96 well plates. Mixed samples were incubated for 20 minutes at room temperature and read at OD_405_ against a standard sample containing a known number of exosomes. Finally, the quantity of exosomes in each sample was calculated using absorbance values from the standard curve created using the standard sample. The final number of exosomes was converted to micrograms, per the manufacturer’s guideline. To characterize exosomes, isolated exosome preparations were re-suspended with PBS and analysed by dynamic light scattering system (Zetasizer Nano, Malvern Instruments Ltd, Worcestershire, UK).

### Mouse cell isolation and culture

Male C57BL/6 based wild type (WT) mice were purchased from the Jackson Laboratory (Bar Harbor, ME, USA) and housed in a specific pathogen-free animal facility (SPF) at Korea Advanced Institute of Science and Technology (KAIST, Daejeon, South Korea). All researchers followed the guidelines of the Care and Use of Laboratory Animal published by NIH. All experimental procedures were approved by the Institutional Animal Care and Use Committee (IACUC) of KAIST. Hepatocytes and HSCs were isolated from mice using collagenase perfusion through the portal vein as previously reported^44^. Isolated hepatocytes were cultured in DMEM with 10% exosome-depleted FBS and 1% penicillin/streptomycin. After 0.4 mM PA treatment for 16 hours, the supernatant was collected and exosomes were isolated. The isolated HSCs were cultured for four days in RPMI with 10% exosome-depleted FBS. Isolated exosomes were added to day 4 HSCs and cultured for 16 hours.

### Transmission electron microscopy (TEM)

Isolated exosomes were re-suspended in 1 mL of PBS and 3 μL of sample was aliquoted into a glow-discharged copper grid covered with carbon film. After 30 seconds, the sample was stained with 3% uranyl acetate and observed using a Tecnai T120 microscope. Images were obtained at 67,000× magnification using an FEI Eagle 4 K × 4 K CCD camera (1.64 Å/pixel, FEI Eindhoven, The Netherlands). Whole exosomes in the images were measured to determine their size.

### Microarray

Total RNA was extracted from exosomes using TRI reagent (Molecular Research Center, Inc., Cincinnati, OH, USA) following the manufacturer’s guidelines. Labelling was performed using 250 ng of total RNA. Each RNA strand was reacted with RNA polymerase for poly-A tailing and ligation was performed with a biotin-labelled 3DNA dendrimer. Reacted RNA strands were hybridized to an Affymetrix GeneChip miRNA 4.0 Array (Affymetrix, Santa Clara, CA, USA) for 18 hours at 48 °C. The chip was washed and stained using an Affymetrix Fluidics Station 450. After amplification, fluorescence signals were detected with an Affymetrix GeneChip Scanner 3000 7 G. The results were analysed with an Agilent scanner and associated software. The expression levels of miRNAs were analysed using Expression Console 1.4.1 (Affymetrix). For normalization of miRNA expression, we performed a quantile normalization using GeneSpring GX 13.1.1 (Agilent technologies, Santa Clara, CA, USA).

### Western blot analysis

Proteins were extracted from isolated exosome with M-PER buffer (Pierce Biotechnology, Rockford, IL, USA). Immunoblots were performed with 30 μg of exosome proteins using antibodies for CD9 and CD63 (Systemic Bioscience). Proteins were separated by electrophoresis through 12% SDS-polyacrylamide gels and then transferred to nitrocellulose membranes. Finally, protein bands were visualized using ECL (Perkin Elmer, Waltham, MA, USA).

### Treatment of LX-2 cells with exosomes

To investigate the interaction between exosomes and HSCs, LX-2 cells were treated with isolated exosomes. LX-2 cells were cultured in high glucose DMEM. Prior to exosome exposure, the FBS-containing medium was removed and LX-2 cells were cultured in 10% exosome-depleted FBS media **(**Systemic Bioscience). LX-2 cells were exposed to 50 and 100 μg of exosomes that were isolated from Huh7 cells that had been treated with or without PA for 16 hours. For repetitive treatment of exosomes, LX-2 cells were treated with exosome every 24 hours for three times. After co-culturing, LX-2 cells were harvested and subjected to quantitative PCR analyses.

### Transfection of miRNA 192

LX-2 cells were seeded in 6-well plates (2 × 10^5^ cells/mL) and allowed to reach a confluency of about 60–80%. Overexpression of miR-192 was achieved by transfection of a miR-192 mimic (50 nM, MC10456; Thermo Fisher Scientific, Waltham, MA, USA) at a concentration of 150 pmol. For negative controls, mirVana miRNA Mimic Negative Control (50 nM, 4464058, Ambion) was used. For cell transfection, Lipofectamine® RNAiMAX (Life technologies, Carlsbad, CA, USA) was used under the guidance of the manufacturer’s instructions. Next, 150 µL of diluted Lipofectamine® RNAiMAX regent and 150 µL of each diluted miRNA-192 or negative control were mixed and incubated at 25 °C for 5 min to generate miRNA-Lipofectamine complexes. Following this, 300 µL of miRNA-Lipofectamine complex was added to the cells and incubated for six hours. After 48 hours of transfection, the target gene level was analysed by quantitative real-time PCR.

### cDNA synthesis and quantitative polymerase chain reaction (PCR)

For analysis of gene expression in LX-2 cells, total RNA was extracted using TRIzol (Ambion, Life Technologies). cDNA was synthesized from isolated RNA using a High Capacity cDNA Reverse Transcription Kit (Applied Biosystems, Foster City, CA, USA) following the manufacturer’s protocol. Quantitative real-time PCR was performed using cDNA and the SYBR green dye (Applied Biosystems). The gene expression levels of alpha smooth muscle actin (α-SMA), transforming growth factor β (TGF-β), and collagenase 1a1 (Col1a1) were compared with GAPDH and change in expression calculated using the ΔΔCt values methods. The sequence of primer is shown in Supplementary Table 3. The expression levels of miRNAs were compared to RNU6B.

### Statistical Analysis

All data are presented as the mean ± SEM. A student’s *t-*test was performed to compare values obtained between the two groups. P values < 0.05 were considered statistically significant.

## Electronic supplementary material

Supplemental data
