# Supplementary material for: Exosomes derived from palmitic acid-treated hepatocytes induce fibrotic activation of hepatic stellate cells
Source: Sci Rep. 2017 Jun 16;7:3710. doi: 10.1038/s41598-017-03389-2 (PMC5473841; doi:10.1038/s41598-017-03389-2)
Supplement: Supplementary file 1 — Supplemental data [file 41598_2017_3389_MOESM1_ESM.doc]

**Supplemental Data**

**Exosomes derived from palmitic acid-treated hepatocytes induce fibrotic activation of hepatic stellate cells**

Young-Sun Lee^1,*^, So Yeon Kim^2,*^, Eunjung Ko^1^, Jun-Hee Lee^3^, Hyon-Seung Yi^4^, Yang Jae Yoo^1^, Jihye Je^1^, Sang Jun Suh^1^, Young Kul Jung^1^, Ji Hoon Kim^1^, Yeon Seok Seo^1^, Hyung Joon Yim^1^, Won-Il Jeong^2,3^, Jong Eun Yeon^1^, Soon Ho Um^1^ & Kwan Soo Byun^1^

^1^Department of Internal Medicine, Korea University College of Medicine, Seoul, Korea. ^2^Lab of Liver Research, Biomedical Science and Engineering Interdisciplinary Program, Korea Advanced Institute of Science and Technology (KAIST), Daejeon 34141, Republic of Korea. ^3^Graduate School of Medical Science and Engineering, Korea Advanced Institute of Science and Technology (KAIST), Daejeon 34141, Republic of Korea. ^4^Department of Internal Medicine, Chungnam National University School of Medicine, Daejeon 305-764, Republic of Korea.

^*^These authors contributed equally to this work.


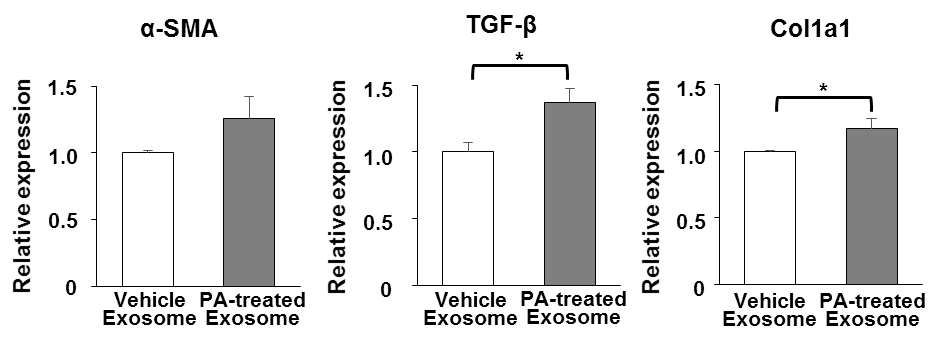


**Supplementary Figure S1. Repetitive treatment of exosomes derived from PA-treated hepatocytes enhance expression of fibrosis markers in HSCs**. LX-2 cells were exposed to exosomes isolated from vehicle-treated Huh7 cells or PA-treated (0.4mM) Huh7 cells. Exosomes, at a concentration of 50 μg/mL, were added to cells every 24 hours for a total tree times. The expression levels of α-SMA, TGF-β, and Col1a1 were then measured in LX-2 cells using real-time PCR. *P < 0.05 compared with the corresponding control.


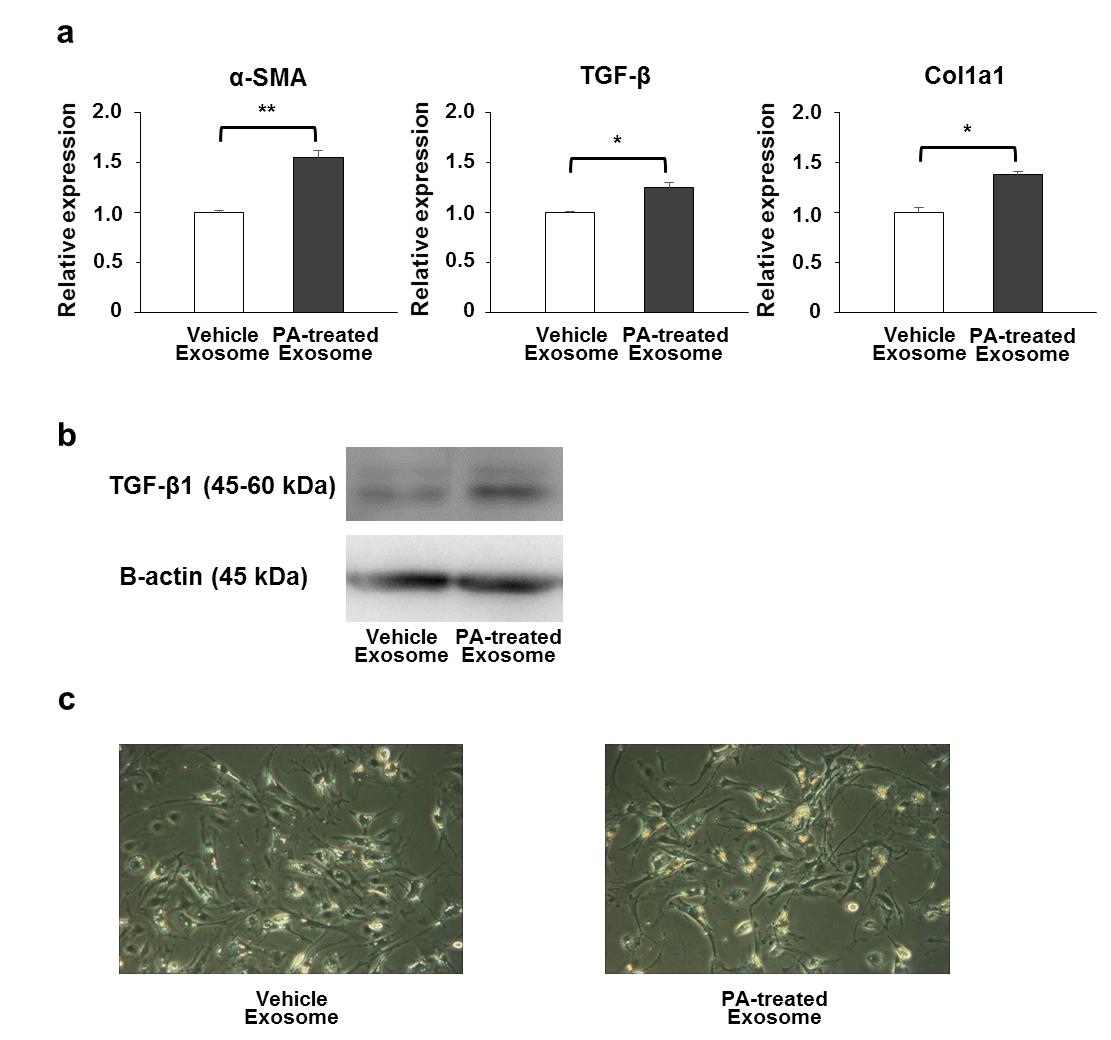


**Supplementary Figure S2. Exosomes derived from PA-treated mouse hepatocytes enhance expression of fibrosis markers in mouse HSCs**. Mouse HSCs were exposed to isolated exosomes from vehicle-treated or PA-treated (0.4mM) mouse hepatocytes. Each isolated exosome preparation was added to HSCs at a concentration of 50 μg/mL. **(a)** The expression levels of α-SMA, TGF-β, and Col1a1 were measured in mouse HSCs using real-time PCR. **(b)** TGF-β protein expression levels were measured in mouse HSCs by Western blot. **c)** HSCs were co-cultured with exosomes from vehicle-treated or PA-treated mouse hepatocytes for 16 hours (original magnification X200). *P < 0.05, **P < 0.01 compared with the corresponding control.

**
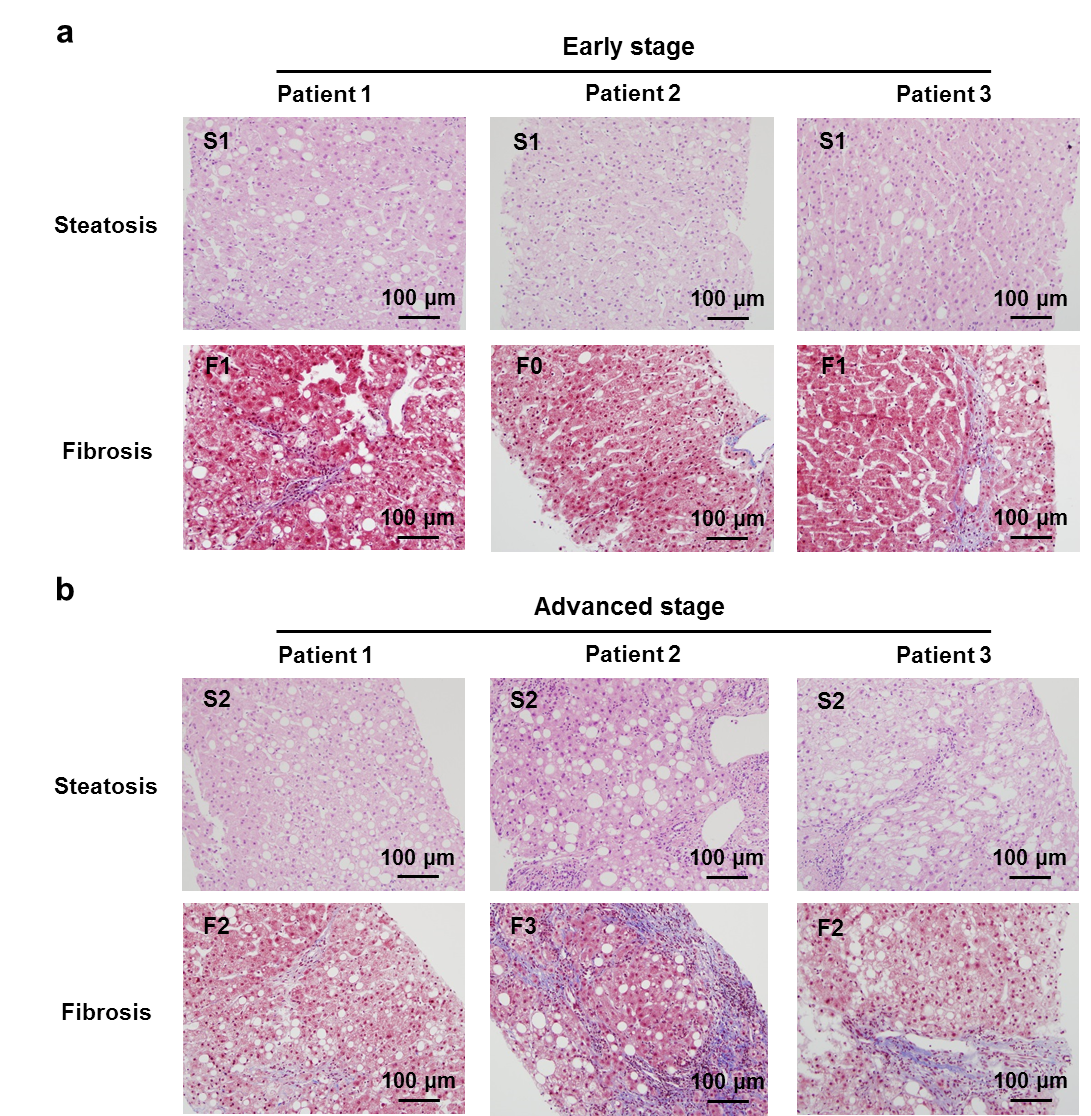
**

**Supplementary Figure S3. Steatosis and fibrosis grades in patients with NAFLD. (a)** Early stage patients showing staining for steatosis and fibrosis. **(b)** Advanced stage patients showing staining for steatosis and fibrosis. The grades are based on the following scoring system: For steatosis, S0, <5%; S1, 5%-33%; S2, >33-66%; S4, >66% and for fibrosis, F0, none; F1, peri-sinusoidal or peri-portal; F2, peri-sinusoidal and portal/peri-portal; F3, bridging fibrosis; F4, cirrhosis. The steatosis grades and fibrosis grades are shown as labels in the left-hand top corner of the images. The bars in each micrograph represent 100μm.

**Supplementary Table S1.** List of miRNAs that showed significantly increased expression in exosomes from PA-treated hepatocytes.

| Name of miRNA | Log ratio^*^ | P-value | Probe Sequence |
| --- | --- | --- | --- |
| hsa-miR-17-5p | 11.01 | <0.01 | CAAAGUGCUUACAGUGCAGGUAG |
| hsa-miR-93-5p | 10.98 | <0.01 | CAAAGUGCUGUUCGUGCAGGUAG |
| hsa-miR-185-5p | 10.81 | <0.01 | UGGAGAGAAAGGCAGUUCCUGA |
| hsa-miR-122-5p | 10.66 | <0.01 | UGGAGUGUGACAAUGGUGUUUG |
| hsa-miR-130b-3p | 10.55 | <0.01 | CAGUGCAAUGAUGAAAGGGCAU |
| hsa-miR-16-5p | 10.45 | <0.01 | UAGCAGCACGUAAAUAUUGGCG |
| hsa-miR-23a-3p | 10.03 | <0.01 | AUCACAUUGCCAGGGAUUUCC |
| hsa-miR-24-3p | 10.01 | <0.01 | UGGCUCAGUUCAGCAGGAACAG |
| hsa-miR-20a-5p | 9.59 | <0.01 | UAAAGUGCUUAUAGUGCAGGUAG |
| hsa-miR-106a-5p | 9.49 | <0.01 | AAAAGUGCUUACAGUGCAGGUAG |
| hsa-miR-107 | 9.46 | <0.01 | AGCAGCAUUGUACAGGGCUAUCA |
| hsa-miR-222-3p | 9.43 | <0.01 | AGCUACAUCUGGCUACUGGGU |
| hsa-miR-210-3p | 9.02 | <0.01 | CUGUGCGUGUGACAGCGGCUGA |
| hsa-miR-221-3p | 9.00 | <0.01 | AGCUACAUUGUCUGCUGGGUUUC |
| hsa-miR-27a-3p | 8.92 | <0.01 | UUCACAGUGGCUAAGUUCCGC |
| hsa-miR-130a-3p | 8.87 | <0.01 | CAGUGCAAUGUUAAAAGGGCAU |
| hsa-miR-103a-3p | 8.68 | <0.01 | AGCAGCAUUGUACAGGGCUAUGA |
| hsa-miR-92a-3p | 8.59 | <0.01 | UAUUGCACUUGUCCCGGCCUGU |
| hsa-miR-345-5p | 8.53 | <0.01 | GCUGACUCCUAGUCCAGGGCUC |
| hsa-miR-378a-3p | 8.32 | <0.01 | ACUGGACUUGGAGUCAGAAGGC |
| hsa-miR-99b-5p | 8.11 | <0.01 | CACCCGUAGAACCGACCUUGCG |
| hsa-miR-532-5p | 7.93 | <0.01 | CAUGCCUUGAGUGUAGGACCGU |
| hsa-miR-191-5p | 7.89 | <0.01 | CAACGGAAUCCCAAAAGCAGCUG |
| hsa-miR-25-3p | 7.74 | 0.02 | CAUUGCACUUGUCUCGGUCUGA |
| hsa-miR-4454 | 7.61 | <0.01 | GGAUCCGAGUCACGGCACCA |
| hsa-miR-320d | 7.54 | <0.01 | AAAAGCUGGGUUGAGAGGA |
| hsa-miR-22-3p | 7.52 | <0.01 | AAGCUGCCAGUUGAAGAACUGU |
| hsa-miR-193a-5p | 7.51 | <0.01 | UGGGUCUUUGCGGGCGAGAUGA |
| hsa-miR-4429 | 7.44 | <0.01 | AAAAGCUGGGCUGAGAGGCG |
| hsa-miR-574-3p | 7.36 | <0.01 | CACGCUCAUGCACACACCCACA |
| hsa-miR-140-3p | 7.27 | <0.01 | UACCACAGGGUAGAACCACGG |
| hsa-miR-146a-5p | 7.23 | 0.02 | UGAGAACUGAAUUCCAUGGGUU |
| hsa-miR-19b-3p | 7.16 | 0.01 | UGUGCAAAUCCAUGCAAAACUGA |
| hsa-miR-320e | 7.07 | <0.01 | AAAGCUGGGUUGAGAAGG |
| hsa-miR-27b-3p | 7.03 | 0.01 | UUCACAGUGGCUAAGUUCUGC |
| hsa-miR-193b-3p | 6.97 | <0.01 | AACUGGCCCUCAAAGUCCCGCU |
| Name of miRNA | Log ratio^*^ | P-value | Probe Sequence |
| hsa-miR-502-3p | 6.92 | <0.01 | AAUGCACCUGGGCAAGGAUUCA |
| hsa-miR-92b-3p | 6.90 | <0.01 | UAUUGCACUCGUCCCGGCCUCC |
| hsa-miR-500a-3p | 6.72 | <0.01 | AUGCACCUGGGCAAGGAUUCUG |
| hsa-miR-23b-3p | 6.70 | <0.01 | AUCACAUUGCCAGGGAUUACC |
| hsa-miR-34a-5p | 6.32 | <0.01 | UGGCAGUGUCUUAGCUGGUUGU |
| hsa-miR-151a-3p | 6.19 | <0.01 | CUAGACUGAAGCUCCUUGAGG |
| hsa-miR-128-3p | 6.08 | <0.01 | UCACAGUGAACCGGUCUCUUU |
| hsa-miR-503-5p | 5.86 | 0.01 | UAGCAGCGGGAACAGUUCUGCAG |
| hsa-miR-20b-5p | 5.77 | 0.02 | CAAAGUGCUCAUAGUGCAGGUAG |
| hsa-miR-425-5p | 5.69 | <0.01 | AAUGACACGAUCACUCCCGUUGA |
| hsa-miR-192-5p | 5.67 | 0.04 | CUGACCUAUGAAUUGACAGCC |
| hsa-miR-339-3p | 5.65 | <0.01 | UGAGCGCCUCGACGACAGAGCCG |
| hsa-miR-15b-5p | 5.62 | 0.01 | UAGCAGCACAUCAUGGUUUACA |
| hsa-miR-30d-5p | 5.62 | 0.01 | UGUAAACAUCCCCGACUGGAAG |
| hsa-miR-501-3p | 5.53 | <0.01 | AAUGCACCCGGGCAAGGAUUCU |
| hsa-miR-423-3p | 5.40 | <0.01 | AGCUCGGUCUGAGGCCCCUCAGU |
| hsa-miR-18a-5p | 5.30 | <0.01 | UAAGGUGCAUCUAGUGCAGAUAG |
| hsa-miR-181b-5p | 5.28 | 0.03 | AACAUUCAUUGCUGUCGGUGGGU |
| hsa-miR-424-3p | 5.05 | <0.01 | CAAAACGUGAGGCGCUGCUAU |
| hsa-miR-378c | 4.96 | <0.01 | ACUGGACUUGGAGUCAGAAGAGUGG |
| hsa-miR-106b-3p | 4.91 | <0.01 | CCGCACUGUGGGUACUUGCUGC |
| hsa-miR-320b | 4.89 | <0.01 | AAAAGCUGGGUUGAGAGGGCAA |
| hsa-miR-151a-5p | 4.80 | <0.01 | UCGAGGAGCUCACAGUCUAGU |
| hsa-miR-106b-5p | 4.74 | 0.01 | UAAAGUGCUGACAGUGCAGAU |
| hsa-miR-320a | 4.63 | <0.01 | AAAAGCUGGGUUGAGAGGGCGA |
| hsa-miR-29a-3p | 4.62 | 0.01 | UAGCACCAUCUGAAAUCGGUUA |
| hsa-miR-181a-5p | 4.38 | <0.01 | AACAUUCAACGCUGUCGGUGAGU |
| hsa-miR-483-5p | 4.33 | 0.01 | AAGACGGGAGGAAAGAAGGGAG |
| hsa-miR-615-3p | 4.30 | <0.01 | UCCGAGCCUGGGUCUCCCUCUU |
| hsa-miR-320c | 4.28 | <0.01 | AAAAGCUGGGUUGAGAGGGU |
| hsa-miR-1307-5p | 4.15 | <0.01 | UCGACCGGACCUCGACCGGCU |
| hsa-miR-324-5p | 4.03 | <0.01 | CGCAUCCCCUAGGGCAUUGGUGU |
| hsa-miR-125a-3p | 3.91 | 0.01 | ACAGGUGAGGUUCUUGGGAGCC |
| hsa-miR-99a-5p | 3.85 | <0.01 | AACCCGUAGAUCCGAUCUUGUG |
| hsa-miR-132-3p | 3.80 | <0.01 | UAACAGUCUACAGCCAUGGUCG |
| hsa-miR-625-5p | 3.71 | <0.01 | AGGGGGAAAGUUCUAUAGUCC |
| hsa-miR-148a-3p | 3.64 | <0.01 | UCAGUGCACUACAGAACUUUGU |
| hsa-miR-125b-2-3p | 3.62 | <0.01 | UCACAAGUCAGGCUCUUGGGAC |
| hsa-miR-660-5p | 3.61 | 0.01 | UACCCAUUGCAUAUCGGAGUUG |
| hsa-miR-331-5p | 3.58 | <0.01 | CUAGGUAUGGUCCCAGGGAUCC |
| Name of miRNA | Log ratio^*^ | P-value | Probe Sequence |
| hsa-miR-378f | 3.55 | <0.01 | ACUGGACUUGGAGCCAGAAG |
| hsa-miR-99b-3p | 3.53 | <0.01 | CAAGCUCGUGUCUGUGGGUCCG |
| hsa-miR-589-5p | 3.47 | <0.01 | UGAGAACCACGUCUGCUCUGAG |
| hsa-miR-125a-5p | 3.44 | <0.01 | UCCCUGAGACCCUUUAACCUGUGA |
| hsa-miR-3615 | 3.41 | <0.01 | UCUCUCGGCUCCUCGCGGCUC |
| hsa-miR-1271-5p | 3.39 | <0.01 | CUUGGCACCUAGCAAGCACUCA |
| hsa-miR-18a-3p | 3.30 | <0.01 | ACUGCCCUAAGUGCUCCUUCUGG |
| hsa-miR-361-5p | 3.24 | <0.01 | UUAUCAGAAUCUCCAGGGGUAC |
| hsa-miR-4306 | 3.23 | <0.01 | UGGAGAGAAAGGCAGUA |
| hsa-miR-21-3p | 3.17 | <0.01 | CAACACCAGUCGAUGGGCUGU |
| hsa-miR-1296-5p | 3.16 | <0.01 | UUAGGGCCCUGGCUCCAUCUCC |
| hsa-miR-330-3p | 3.12 | <0.01 | GCAAAGCACACGGCCUGCAGAGA |
| hsa-miR-152-3p | 3.03 | <0.01 | UCAGUGCAUGACAGAACUUGG |
| hsa-miR-1269a | 2.97 | <0.01 | CUGGACUGAGCCGUGCUACUGG |
| hsa-miR-652-3p | 2.96 | <0.01 | AAUGGCGCCACUAGGGUUGUG |
| hsa-miR-151b | 2.93 | <0.01 | UCGAGGAGCUCACAGUCU |
| hsa-miR-1269b | 2.92 | <0.01 | CUGGACUGAGCCAUGCUACUGG |
| hsa-miR-3679-5p | 2.85 | <0.01 | UGAGGAUAUGGCAGGGAAGGGGA |
| hsa-miR-188-5p | 2.83 | <0.01 | CAUCCCUUGCAUGGUGGAGGG |
| hsa-miR-422a | 2.81 | <0.01 | ACUGGACUUAGGGUCAGAAGGC |
| hsa-miR-26a-5p | 2.75 | 0.04 | UUCAAGUAAUCCAGGAUAGGCU |
| hsa-miR-3660 | 2.69 | <0.01 | ACUGACAGGAGAGCAUUUUGA |
| hsa-miR-7854-3p | 2.67 | <0.01 | UGAGGUGACCGCAGAUGGGAA |
| hsa-miR-134-5p | 2.67 | <0.01 | UGUGACUGGUUGACCAGAGGGG |
| hsa-miR-378i | 2.67 | <0.01 | ACUGGACUAGGAGUCAGAAGG |
| hsa-miR-425-3p | 2.64 | <0.01 | AUCGGGAAUGUCGUGUCCGCCC |
| hsa-miR-138-5p | 2.57 | <0.01 | AGCUGGUGUUGUGAAUCAGGCCG |
| hsa-miR-4668-5p | 2.53 | <0.01 | AGGGAAAAAAAAAAGGAUUUGUC |
| hsa-miR-324-3p | 2.52 | 0.02 | ACUGCCCCAGGUGCUGCUGG |
| hsa-miR-1301-3p | 2.52 | <0.01 | UUGCAGCUGCCUGGGAGUGACUUC |
| hsa-miR-1285-3p | 2.48 | <0.01 | UCUGGGCAACAAAGUGAGACCU |
| hsa-let-7e-5p | 2.47 | <0.01 | UGAGGUAGGAGGUUGUAUAGUU |
| hsa-miR-378d | 2.44 | <0.01 | ACUGGACUUGGAGUCAGAAA |
| hsa-miR-532-3p | 2.43 | <0.01 | CCUCCCACACCCAAGGCUUGCA |
| hsa-miR-616-3p | 2.42 | <0.01 | AGUCAUUGGAGGGUUUGAGCAG |
| hsa-miR-550a-3-5p | 2.29 | <0.01 | AGUGCCUGAGGGAGUAAGAG |
| hsa-miR-1304-3p | 2.23 | <0.01 | UCUCACUGUAGCCUCGAACCCC |
| hsa-miR-148b-3p | 2.21 | 0.01 | UCAGUGCAUCACAGAACUUUGU |
| hsa-miR-23b-5p | 2.18 | <0.01 | UGGGUUCCUGGCAUGCUGAUUU |
| hsa-miR-4497 | 2.16 | 0.01 | CUCCGGGACGGCUGGGC |
| Name of miRNA | Log ratio^*^ | P-value | Probe Sequence |
| hsa-miR-193a-3p | 2.13 | <0.01 | AACUGGCCUACAAAGUCCCAGU |
| hsa-miR-378g | 2.12 | <0.01 | ACUGGGCUUGGAGUCAGAAG |
| hsa-miR-18b-5p | 2.08 | <0.01 | UAAGGUGCAUCUAGUGCAGUUAG |
| hsa-miR-1295a | 2.07 | <0.01 | UUAGGCCGCAGAUCUGGGUGA |
| hsa-miR-143-3p | 2.01 | <0.01 | UGAGAUGAAGCACUGUAGCUC |
| hsa-miR-1254 | 1.99 | <0.01 | AGCCUGGAAGCUGGAGCCUGCAGU |
| hsa-miR-500b-3p | 1.98 | <0.01 | GCACCCAGGCAAGGAUUCUG |
| hsa-miR-3613-3p | 1.96 | 0.01 | ACAAAAAAAAAAGCCCAACCCUUC |
| hsa-miR-619-5p | 1.95 | <0.01 | GCUGGGAUUACAGGCAUGAGCC |
| hsa-miR-491-5p | 1.92 | <0.01 | AGUGGGGAACCCUUCCAUGAGG |
| hsa-miR-200b-5p | 1.91 | <0.01 | CAUCUUACUGGGCAGCAUUGGA |
| hsa-miR-877-5p | 1.90 | 0.02 | GUAGAGGAGAUGGCGCAGGG |
| hsa-miR-30a-5p | 1.86 | 0.04 | UGUAAACAUCCUCGACUGGAAG |
| hsa-miR-671-5p | 1.82 | 0.02 | AGGAAGCCCUGGAGGGGCUGGAG |
| hsa-miR-3909 | 1.80 | 0.02 | UGUCCUCUAGGGCCUGCAGUCU |
| hsa-miR-3131 | 1.77 | <0.01 | UCGAGGACUGGUGGAAGGGCCUU |
| hsa-miR-1273d | 1.77 | <0.01 | GAACCCAUGAGGUUGAGGCUGCAGU |
| hsa-miR-28-5p | 1.75 | 0.01 | AAGGAGCUCACAGUCUAUUGAG |
| hsa-miR-642a-3p | 1.73 | 0.01 | AGACACAUUUGGAGAGGGAACC |
| hsa-miR-183-5p | 1.72 | 0.02 | UAUGGCACUGGUAGAAUUCACU |
| hsa-miR-6068 | 1.71 | 0.01 | CCUGCGAGUCUCCGGCGGUGG |
| hsa-miR-34a-3p | 1.68 | 0.03 | CAAUCAGCAAGUAUACUGCCCU |
| hsa-miR-34c-3p | 1.65 | <0.01 | AAUCACUAACCACACGGCCAGG |
| hsa-miR-3944-5p | 1.64 | 0.02 | UGUGCAGCAGGCCAACCGAGA |
| hsa-miR-3194-5p | 1.62 | 0.02 | GGCCAGCCACCAGGAGGGCUG |
| hsa-miR-331-3p | 1.62 | <0.01 | GCCCCUGGGCCUAUCCUAGAA |
| hsa-miR-182-5p | 1.60 | <0.01 | UUUGGCAAUGGUAGAACUCACACU |
| hsa-miR-1273f | 1.57 | <0.01 | GGAGAUGGAGGUUGCAGUG |
| hsa-miR-15a-5p | 1.50 | 0.04 | UAGCAGCACAUAAUGGUUUGUG |
| hsa-miR-3654 | 1.48 | <0.01 | GACUGGACAAGCUGAGGAA |
| hsa-miR-17-3p | 1.48 | 0.01 | ACUGCAGUGAAGGCACUUGUAG |
| hsa-miR-1273h-5p | 1.45 | <0.01 | CUGGGAGGUCAAGGCUGCAGU |
| hsa-miR-93-3p | 1.44 | <0.01 | ACUGCUGAGCUAGCACUUCCCG |
| hsa-miR-671-3p | 1.43 | <0.01 | UCCGGUUCUCAGGGCUCCACC |
| hsa-miR-3928-3p | 1.41 | 0.01 | GGAGGAACCUUGGAGCUUCGGC |
| hsa-miR-8075 | 1.41 | 0.03 | UGCUGAUGGCAGAUGUCGGGUCUG |
| hsa-miR-224-3p | 1.41 | 0.03 | AAAAUGGUGCCCUAGUGACUACA |
| hsa-miR-2277-3p | 1.36 | 0.01 | UGACAGCGCCCUGCCUGGCUC |
| hsa-miR-942-3p | 1.35 | 0.01 | CACAUGGCCGAAACAGAGAAGU |
| hsa-miR-7706 | 1.35 | 0.01 | UGAAGCGCCUGUGCUCUGCCGAGA |
| Name of miRNA | Log ratio^*^ | P-value | Probe Sequence |
| hsa-miR-3682-3p | 1.25 | 0.03 | UGAUGAUACAGGUGGAGGUAG |
| hsa-let-7c-5p | 1.23 | 0.04 | UGAGGUAGUAGGUUGUAUGGUU |
| hsa-miR-3138 | 1.21 | <0.01 | UGUGGACAGUGAGGUAGAGGGAGU |
| hsa-miR-1266-5p | 1.19 | 0.01 | CCUCAGGGCUGUAGAACAGGGCU |
| hsa-miR-4652-5p | 1.16 | 0.04 | AGGGGACUGGUUAAUAGAACUA |
| hsa-miR-6734-5p | 1.15 | 0.01 | UUGAGGGGAGAAUGAGGUGGAGA |
| hsa-miR-212-3p | 1.12 | <0.01 | UAACAGUCUCCAGUCACGGCC |
| hsa-miR-602 | 1.09 | <0.01 | GACACGGGCGACAGCUGCGGCCC |
| hsa-miR-7975 | 1.07 | 0.01 | AUCCUAGUCACGGCACCA |
| hsa-miR-185-3p | 1.06 | <0.01 | AGGGGCUGGCUUUCCUCUGGUC |
| hsa-miR-1226-3p | 1.04 | <0.01 | UCACCAGCCCUGUGUUCCCUAG |
| hsa-miR-4417 | 1.04 | 0.01 | GGUGGGCUUCCCGGAGGG |
| hsa-miR-4486 | 1.03 | 0.04 | GCUGGGCGAGGCUGGCA |
| hsa-miR-4539 | 1.02 | <0.01 | GCUGAACUGGGCUGAGCUGGGC |
| hsa-miR-1247-5p | 1.01 | 0.02 | ACCCGUCCCGUUCGUCCCCGGA |
| hsa-miR-3157-5p | 1.01 | 0.01 | UUCAGCCAGGCUAGUGCAGUCU |
| hsa-miR-3663-3p | 1.01 | <0.01 | UGAGCACCACACAGGCCGGGCGC |

*PA treated group *vs.* vehicle treated group

**Supplementary Table S2.** List of miRNAs that showed significantly decreased expression in exosomes from PA-treated hepatocytes.

| Name of miRNA | Log ratio^*^ | P-value | Probe Sequence |
| --- | --- | --- | --- |
| hsa-miR-6825-5p | -7.64 | 0.00 | UGGGGAGGUGUGGAGUCAGCAU |
| hsa-miR-4689 | -7.15 | 0.00 | UUGAGGAGACAUGGUGGGGGCC |
| hsa-miR-4463 | -6.50 | 0.00 | GAGACUGGGGUGGGGCC |
| hsa-miR-6765-5p | -6.43 | 0.00 | GUGAGGCGGGGCCAGGAGGGUGUGU |
| hsa-miR-6756-5p | -6.31 | 0.00 | AGGGUGGGGCUGGAGGUGGGGCU |
| hsa-miR-1228-5p | -5.83 | 0.00 | GUGGGCGGGGGCAGGUGUGUG |
| hsa-miR-6716-5p | -5.82 | 0.00 | UGGGAAUGGGGGUAAGGGCC |
| hsa-miR-4433b-3p | -5.67 | 0.00 | CAGGAGUGGGGGGUGGGACGU |
| hsa-miR-6749-5p | -5.51 | 0.00 | UCGGGCCUGGGGUUGGGGGAGC |
| hsa-miR-4532 | -5.49 | 0.00 | CCCCGGGGAGCCCGGCG |
| hsa-miR-4459 | -5.47 | 0.00 | CCAGGAGGCGGAGGAGGUGGAG |
| hsa-miR-92b-5p | -5.40 | 0.00 | AGGGACGGGACGCGGUGCAGUG |
| hsa-miR-4632-5p | -5.25 | 0.00 | GAGGGCAGCGUGGGUGUGGCGGA |
| hsa-miR-6743-5p | -5.21 | 0.00 | AAGGGGCAGGGACGGGUGGCCC |
| hsa-miR-4651 | -5.02 | 0.00 | CGGGGUGGGUGAGGUCGGGC |
| hsa-miR-4433-3p | -4.79 | 0.00 | ACAGGAGUGGGGGUGGGACAU |
| hsa-miR-6722-3p | -4.65 | 0.00 | UGCAGGGGUCGGGUGGGCCAGG |
| hsa-miR-4758-5p | -4.62 | 0.00 | GUGAGUGGGAGCCGGUGGGGCUG |
| hsa-miR-6821-5p | -4.51 | 0.00 | GUGCGUGGUGGCUCGAGGCGGGG |
| hsa-miR-4707-5p | -4.47 | 0.00 | GCCCCGGCGCGGGCGGGUUCUGG |
| hsa-miR-3921 | -4.44 | 0.01 | UCUCUGAGUACCAUAUGCCUUGU |
| hsa-miR-6849-5p | -4.42 | 0.00 | GAGUGGAUAGGGGAGUGUGUGGA |
| hsa-miR-6803-5p | -4.42 | 0.00 | CUGGGGGUGGGGGGCUGGGCGU |
| hsa-miR-4281 | -4.36 | 0.00 | GGGUCCCGGGGAGGGGGG |
| hsa-miR-1281 | -4.28 | 0.02 | UCGCCUCCUCCUCUCCC |
| hsa-miR-6824-5p | -4.27 | 0.00 | GUAGGGGAGGUUGGGCCAGGGA |
| hsa-miR-6794-5p | -4.27 | 0.00 | CAGGGGGACUGGGGGUGAGC |
| hsa-miR-6789-5p | -4.25 | 0.00 | GUAGGGGCGUCCCGGGCGCGCGGG |
| hsa-miR-4763-3p | -4.23 | 0.00 | AGGCAGGGGCUGGUGCUGGGCGGG |
| hsa-miR-4428 | -4.03 | 0.01 | CAAGGAGACGGGAACAUGGAGC |
| hsa-miR-4749-5p | -3.98 | 0.01 | UGCGGGGACAGGCCAGGGCAUC |
| Name of miRNA | Log ratio^*^ | P-value | Probe Sequence |
| hsa-miR-6779-5p | -3.93 | 0.00 | CUGGGAGGGGCUGGGUUUGGC |
| hsa-miR-4701-3p | -3.91 | 0.03 | AUGGGUGAUGGGUGUGGUGU |
| hsa-miR-3621 | -3.91 | 0.00 | CGCGGGUCGGGGUCUGCAGG |
| hsa-miR-6816-5p | -3.89 | 0.00 | UGGGGCGGGGCAGGUCCCUGC |
| hsa-miR-7107-5p | -3.84 | 0.00 | UCGGCCUGGGGAGGAGGAAGGG |
| hsa-miR-5787 | -3.82 | 0.00 | GGGCUGGGGCGCGGGGAGGU |
| hsa-miR-1268b | -3.76 | 0.01 | CGGGCGUGGUGGUGGGGGUG |
| hsa-miR-4449 | -3.76 | 0.01 | CGUCCCGGGGCUGCGCGAGGCA |
| hsa-miR-6798-5p | -3.71 | 0.00 | CCAGGGGGAUGGGCGAGCUUGGG |
| hsa-miR-6775-5p | -3.67 | 0.00 | UCGGGGCAUGGGGGAGGGAGGCUGG |
| hsa-miR-3141 | -3.67 | 0.00 | GAGGGCGGGUGGAGGAGGA |
| hsa-miR-4507 | -3.63 | 0.00 | CUGGGUUGGGCUGGGCUGGG |
| hsa-miR-3656 | -3.62 | 0.00 | GGCGGGUGCGGGGGUGG |
| hsa-miR-3620-5p | -3.61 | 0.00 | GUGGGCUGGGCUGGGCUGGGCC |
| hsa-miR-6741-5p | -3.60 | 0.00 | GUGGGUGCUGGUGGGAGCCGUG |
| hsa-miR-3940-5p | -3.56 | 0.01 | GUGGGUUGGGGCGGGCUCUG |
| hsa-miR-3178 | -3.55 | 0.01 | GGGGCGCGGCCGGAUCG |
| hsa-miR-7844-5p | -3.53 | 0.00 | AAAACUAGGACUGUGUGGUGUA |
| hsa-miR-6819-5p | -3.52 | 0.00 | UUGGGGUGGAGGGCCAAGGAGC |
| hsa-miR-149-3p | -3.48 | 0.00 | AGGGAGGGACGGGGGCUGUGC |
| hsa-miR-328-5p | -3.47 | 0.00 | GGGGGGGCAGGAGGGGCUCAGGG |
| hsa-miR-1469 | -3.42 | 0.00 | CUCGGCGCGGGGCGCGGGCUCC |
| hsa-miR-595 | -3.42 | 0.00 | GAAGUGUGCCGUGGUGUGUCU |
| hsa-miR-150-3p | -3.38 | 0.00 | CUGGUACAGGCCUGGGGGACAG |
| hsa-miR-762 | -3.34 | 0.00 | GGGGCUGGGGCCGGGGCCGAGC |
| hsa-miR-6861-5p | -3.30 | 0.02 | ACUGGGUAGGUGGGGCUCCAGG |
| hsa-miR-6127 | -3.14 | 0.02 | UGAGGGAGUGGGUGGGAGG |
| hsa-miR-6858-5p | -3.06 | 0.00 | GUGAGGAGGGGCUGGCAGGGAC |
| hsa-miR-6787-5p | -2.95 | 0.00 | UGGCGGGGGUAGAGCUGGCUGC |
| hsa-miR-3064-5p | -2.92 | 0.01 | UCUGGCUGUUGUGGUGUGCAA |
| hsa-miR-6785-5p | -2.92 | 0.00 | UGGGAGGGCGUGGAUGAUGGUG |
| hsa-miR-1275 | -2.91 | 0.00 | GUGGGGGAGAGGCUGUC |
| hsa-miR-6786-5p | -2.89 | 0.00 | GCGGUGGGGCCGGAGGGGCGU |
| hsa-miR-5196-5p | -2.85 | 0.01 | AGGGAAGGGGACGAGGGUUGGG |
| hsa-miR-4687-3p | -2.84 | 0.00 | UGGCUGUUGGAGGGGGCAGGC |
| Name of miRNA | Log ratio^*^ | P-value | Probe Sequence |
| hsa-miR-1227-5p | -2.82 | 0.00 | GUGGGGCCAGGCGGUGG |
| hsa-miR-4649-5p | -2.78 | 0.00 | UGGGCGAGGGGUGGGCUCUCAGAG |
| hsa-miR-6870-5p | -2.75 | 0.00 | UGGGGGAGAUGGGGGUUGA |
| hsa-miR-6769b-5p | -2.75 | 0.00 | UGGUGGGUGGGGAGGAGAAGUGC |
| hsa-miR-6087 | -2.73 | 0.00 | UGAGGCGGGGGGGCGAGC |
| hsa-miR-4741 | -2.73 | 0.00 | CGGGCUGUCCGGAGGGGUCGGCU |
| hsa-miR-6791-5p | -2.72 | 0.00 | CCCCUGGGGCUGGGCAGGCGGA |
| hsa-miR-7845-5p | -2.66 | 0.00 | AAGGGACAGGGAGGGUCGUGG |
| hsa-miR-6776-5p | -2.57 | 0.04 | UCUGGGUGCAGUGGGGGUU |
| hsa-miR-296-3p | -2.55 | 0.00 | GAGGGUUGGGUGGAGGCUCUCC |
| hsa-miR-297 | -2.53 | 0.00 | AUGUAUGUGUGCAUGUGCAUG |
| hsa-miR-6812-5p | -2.49 | 0.01 | AUGGGGUGAGAUGGGGAGGAGCAGC |
| hsa-miR-4750-5p | -2.47 | 0.02 | CUCGGGCGGAGGUGGUUGAGUG |
| hsa-miR-6089 | -2.44 | 0.00 | GGAGGCCGGGGUGGGGCGGGGCGG |
| hsa-miR-7108-5p | -2.39 | 0.00 | GUGUGGCCGGCAGGCGGGUGG |
| hsa-miR-1228-3p | -2.39 | 0.00 | UCACACCUGCCUCGCCCCCC |
| hsa-miR-937-5p | -2.37 | 0.00 | GUGAGUCAGGGUGGGGCUGG |
| hsa-miR-3148 | -2.36 | 0.00 | UGGAAAAAACUGGUGUGUGCUU |
| hsa-miR-1343-5p | -2.36 | 0.01 | UGGGGAGCGGCCCCCGGGUGGG |
| hsa-miR-4270 | -2.35 | 0.03 | UCAGGGAGUCAGGGGAGGGC |
| hsa-miR-6724-5p | -2.33 | 0.00 | CUGGGCCCGCGGCGGGCGUGGGG |
| hsa-miR-663a | -2.32 | 0.01 | AGGCGGGGCGCCGCGGGACCGC |
| hsa-miR-638 | -2.27 | 0.00 | AGGGAUCGCGGGCGGGUGGCGGCCU |
| hsa-miR-7106-5p | -2.27 | 0.00 | UGGGAGGAGGGGAUCUUGGG |
| hsa-miR-2861 | -2.25 | 0.00 | GGGGCCUGGCGGUGGGCGG |
| hsa-miR-940 | -2.25 | 0.00 | AAGGCAGGGCCCCCGCUCCCC |
| hsa-miR-6845-5p | -2.23 | 0.01 | CGGGGCCAGAGCAGAGAGC |
| hsa-miR-642b-3p | -2.23 | 0.02 | AGACACAUUUGGAGAGGGACCC |
| hsa-miR-8072 | -2.21 | 0.01 | GGCGGCGGGGAGGUAGGCAG |
| hsa-miR-1237-5p | -2.17 | 0.01 | CGGGGGCGGGGCCGAAGCGCG |
| hsa-miR-1229-5p | -2.17 | 0.00 | GUGGGUAGGGUUUGGGGGAGAGCG |
| hsa-miR-6763-5p | -2.11 | 0.01 | CUGGGGAGUGGCUGGGGAG |
| hsa-miR-4739 | -2.10 | 0.00 | AAGGGAGGAGGAGCGGAGGGGCCCU |
| hsa-miR-6805-5p | -2.09 | 0.03 | UAGGGGGCGGCUUGUGGAGUGU |
| hsa-miR-6848-3p | -2.09 | 0.01 | GUGGUCUCUUGGCCCCCAG |
| Name of miRNA | Log ratio^*^ | Log ratio^*^ | Probe Sequence |
| hsa-miR-6848-5p | -2.07 | 0.02 | UGGGGGCUGGGAUGGGCCAUGGU |
| hsa-miR-3622a-3p | -2.03 | 0.00 | UCACCUGACCUCCCAUGCCUGU |
| hsa-miR-6893-5p | -2.02 | 0.02 | CAGGCAGGUGUAGGGUGGAGC |
| hsa-miR-1224-5p | -2.01 | 0.01 | GUGAGGACUCGGGAGGUGG |
| hsa-miR-4698 | -2.01 | 0.01 | UCAAAAUGUAGAGGAAGACCCCA |
| hsa-miR-4674 | -1.98 | 0.03 | CUGGGCUCGGGACGCGCGGCU |
| hsa-miR-6132 | -1.96 | 0.00 | AGCAGGGCUGGGGAUUGCA |
| hsa-miR-4508 | -1.94 | 0.01 | GCGGGGCUGGGCGCGCG |
| hsa-miR-1207-5p | -1.91 | 0.03 | UGGCAGGGAGGCUGGGAGGGG |
| hsa-miR-197-5p | -1.85 | 0.01 | CGGGUAGAGAGGGCAGUGGGAGG |
| hsa-miR-6088 | -1.82 | 0.00 | AGAGAUGAAGCGGGGGGGCG |
| hsa-let-7b-5p | -1.80 | 0.03 | UGAGGUAGUAGGUUGUGUGGUU |
| hsa-miR-4745-5p | -1.71 | 0.01 | UGAGUGGGGCUCCCGGGACGGCG |
| hsa-let-7f-1-3p | -1.68 | 0.03 | CUAUACAAUCUAUUGCCUUCCC |
| hsa-miR-8069 | -1.63 | 0.01 | GGAUGGUUGGGGGCGGUCGGCGU |
| hsa-miR-1343-3p | -1.62 | 0.00 | CUCCUGGGGCCCGCACUCUCGC |
| hsa-miR-1225-5p | -1.62 | 0.00 | GUGGGUACGGCCCAGUGGGGGG |
| hsa-miR-1260b | -1.60 | 0.05 | AUCCCACCACUGCCACCAU |
| hsa-miR-4787-5p | -1.59 | 0.00 | GCGGGGGUGGCGGCGGCAUCCC |
| hsa-miR-7704 | -1.54 | 0.02 | CGGGGUCGGCGGCGACGUG |
| hsa-miR-32-3p | -1.53 | 0.04 | CAAUUUAGUGUGUGUGAUAUUU |
| hsa-miR-4498 | -1.51 | 0.02 | UGGGCUGGCAGGGCAAGUGCUG |
| hsa-miR-466 | -1.50 | 0.01 | AUACACAUACACGCAACACACAU |
| hsa-miR-2114-3p | -1.47 | 0.01 | CGAGCCUCAAGCAAGGGACUU |
| hsa-miR-6090 | -1.47 | 0.00 | GGGGAGCGAGGGGCGGGGC |
| hsa-miR-1908-5p | -1.47 | 0.00 | CGGCGGGGACGGCGAUUGGUC |
| hsa-miR-4517 | -1.43 | 0.04 | AAAUAUGAUGAAACUCACAGCUGAG |
| hsa-miR-6831-5p | -1.40 | 0.03 | UAGGUAGAGUGUGAGGAGGAGGUC |
| hsa-miR-4750-3p | -1.31 | 0.01 | CCUGACCCACCCCCUCCCGCAG |
| hsa-miR-4687-5p | -1.30 | 0.04 | CAGCCCUCCUCCCGCACCCAAA |
| hsa-miR-5094 | -1.27 | 0.04 | AAUCAGUGAAUGCCUUGAACCU |
| hsa-miR-7150 | -1.24 | 0.00 | CUGGCAGGGGGAGAGGUA |
| hsa-miR-5692b | -1.24 | 0.03 | AAUAAUAUCACAGUAGGUGU |
| hsa-miR-4298 | -1.21 | 0.03 | CUGGGACAGGAGGAGGAGGCAG |
| hsa-miR-6836-3p | -1.19 | 0.03 | AUGCCUCCCCCGGCCCCGCAG |
| Name of miRNA | Log ratio^*^ | P-value | Probe Sequence |
| hsa-miR-4466 | -1.15 | 0.00 | GGGUGCGGGCCGGCGGGG |
| hsa-miR-7154-5p | -1.14 | 0.00 | UUCAUGAACUGGGUCUAGCUUGG |
| hsa-miR-1182 | -1.13 | 0.00 | GAGGGUCUUGGGAGGGAUGUGAC |
| hsa-miR-4690-5p | -1.03 | 0.03 | GAGCAGGCGAGGCUGGGCUGAA |
| hsa-miR-548j-5p | -1.01 | 0.00 | AAAAGUAAUUGCGGUCUUUGGU |

*PA treated group *vs.* vehicle treated group

**Supplementary Table S3.** List of primer sequence that used in real-time PCR analysis

| **Primer name** | **Forward (5’-3’)** | **Reverse (5’-3’)** |
| --- | --- | --- |
| **Human** | | |
| α-SMA | TGCCTTGGTGTGTGACAATG | TTGTCCCATTCCCAC CATCA |
| TGF-β | CAGCAGGGATAACAC ACTGC | CATGAGAAGCAGGAAAGGCC |
| Col1a1 | TGACCTCAAGATGTGCCACT | ACCAGTCTCCATGTTGCAGA |
| GAPDH | AGCCACATCGCTCAGACAC | GCCCAATACGACCAAATCC |
| **Mouse** | | |
| α-SMA | CTGACAGAGGCACCACTGAA | GAAGGAATAGCCACGCTCAG |
| TGF-β | TGGTTGTAGAGGGCAAGGAC | TTGCTTCAGCTCCACAGAGA |
| Col1a1 | TCCTCCAGGGATCCAACGA | GGCAGGCGGGAGGTCTT |
| β-actin | GTTACCAACTGGGACGAC | CTCAAACATGATCTGGGTCA |
